# A regulator of G protein signaling 5 marked subpopulation of vascular smooth muscle cells is lost during vascular disease

**DOI:** 10.1371/journal.pone.0265132

**Published:** 2022-03-23

**Authors:** Ya-Kun Gao, Rui-Juan Guo, Xin Xu, Xiao-Fu Huang, Yu Song, Dan-Dan Zhang, Ning Chen, Xiao-Wei Wang, Chen-Xi Liang, Peng Kong, Mei Han

**Affiliations:** 1 Department of Biochemistry and Molecular Biology, College of Basic Medicine, Key Laboratory of Neural and Vascular Biology of Ministry of Education, Key Laboratory of Medical Biotechnology of Hebei Province, Hebei Medical University, Shijiazhuang, China; 2 Laboratory of Lipid Metabolism, Institute of Basic Medicine, Hebei Medical University, Shijiazhuang, China; Max Delbruck Centrum fur Molekulare Medizin Berlin Buch, GERMANY

## Abstract

Vascular smooth muscle cell (VSMC) subpopulations relevant to vascular disease and injury repair have been depicted in healthy vessels and atherosclerosis profiles. However, whether VSMC subpopulation associated with vascular homeostasis exists in the healthy artery and how are their nature and fate in vascular remodeling remains elusive. Here, using single-cell RNA-sequencing (scRNA-seq) to detect VSMC functional heterogeneity in an unbiased manner, we showed that VSMC subpopulations in healthy artery presented transcriptome diversity and that there was significant heterogeneity in differentiation state and development within each subpopulation. Notably, we detected an independent subpopulation of VSMCs that highly expressed regulator of G protein signaling 5 (RGS5), upregulated the genes associated with inhibition of cell proliferation and construction of cytoskeleton compared with the general subpopulation, and mainly enriched in descending aorta. Additionally, the proportion of RGS5^high^ VSMCs was markedly decreased or almost disappeared in the vascular tissues of neointimal formation, abdominal aortic aneurysm and atherosclerosis. Specific spatiotemporal characterization of RGS5^high^ VSMC subpopulation suggested that this subpopulation was implicated in vascular homeostasis. Together, our analyses identify homeostasis-relevant transcriptional signatures of VSMC subpopulations in healthy artery, which may explain the regional vascular resistance to atherosclerosis at some extent.

## Introduction

Despite decades of research and drug development, the unrelenting threat by vascular disorders to human health has motivated the search for renewed knowledge of the vascular biology. Vascular smooth muscle cell (VSMC) heterogeneity emerged as a major focus in mechanistic studies of vascular homeostasis and remodeling [1–3]. VSMCs within healthy vessels are quiescent and characterized by the expression of contractile proteins including smooth muscle (SM) 22α, SM α-actin, SM myosin heavy chain (SMMHC), H1-calponin, smoothelin and caldesmon [4]. However, VSMCs yet retain remarkable plasticity and can dedifferentiate from a contractile state to a synthetic state in response to vascular injury, along with increased extracellular matrix production, migration and proliferation [5, 6]. As a heterogeneous population, VSMCs in healthy artery display dramatic differences in morphology [7], growth characteristics [8], expression pattern of specific marker genes [9] and responses to important signaling pathways [10, 11], indicating that specific VSMC subpopulations relevant to vascular homeostasis and particular disease may exist within healthy artery [12]. It has been hypothesized that VSMCs displaying different levels of plasticity co-exist within the healthy vessel wall [6] and might contribute to the non-random disease susceptibility of different vascular beds. Recent studies using single cell RNA sequencing (scRNA-seq) have depicted some specific VSMC subpopulations relevant to vascular disease and injury repair, such as Klf4^+^ [13], Lgals3^+^ [14], Sca1^+^ [1], TCF21^+^ and Tnfrsf11b^+^ [15] VSMCs of in healthy artery and atherosclerosis profiles. However, whether VSMC subpopulation associated with vascular homeostasis exists in the healthy vessel wall and what are the characterization of the particular VSMC subpopulations remains elusive.

Here, we demonstrated that VSMC subpopulations presented transcriptome diversity and remarkable differentiation and development heterogeneity existed within each subpopulation. Furthermore, we identified that an independent regulator of G protein signaling 5 (RGS5) marked VSMC subpopulation preferentially distributed in the descending aorta compared to other artery regions, especially atherosclerosis-prone regions, and demonstrated that RGS5^high^ VSMC subpopulation reduced or even disappeared in vascular disease. These detailing findings may be beneficial to the development of therapeutics to target for VSMCs-derived diseases.

## Materials and methods

### Animal models

All animal procedures conformed to the Guide for the Care and Use of Laboratory Animals published by the US National Institutes of Health (NIH Publication, 8th Edition, 2011) and approved by the Institutional Animal Care and Use Committee of Hebei Medical University. C57BL/6J and *Ldlr*^*−/−*^ mice were purchased from Charles River. All mice were housed in a specific pathogen-free environment under a 12h/12h light-dark cycle and fed rodent diet at free. Ten- to twelve- week-old male C57BL/6J mice were anaesthetized using 2.5~3% isoflurane by inhalation. The left common carotid artery was tied firmly with one knot using 6–0 silk suture just below the bifurcation point and animals sacrificed at 28th day after surgery. The osmotic mini-pump (ALZET) delivering AngII (1μg/kg/min; Sigma) was implanted subcutaneously in the dorsum of the neck for 28 d to induce abdominal aortic aneurysm. Six- to eight- week-old male *Ldlr*^*−/−*^ mice were fed a high-cholesterol diet (1.25% cholesterol and 40% calories from fat) for 12 weeks to develop atherosclerosis model.

### Analysis of cryosections

In ligation model, the vascular samples were taken from the bifurcation (the place of ligature) of the common carotid artery to 0.5 cm below it. In abdominal aortic aneurysm model, the suprarenal aorta with a 50% or greater increase in the external width and the same site of control arteries were separated. Vascular tissue samples were fixed in 4% paraformaldehyde in PBS for 1 h, followed by overnight in 30% sucrose in PBS at 4°C. OCT-embedded common carotid artery tissues were sequentially sliced (thickness: 10 μm) with freezing microtome from the ligation site, and 50 pieces were sequentially sliced. Sections from the same distance from the ligation site in each experimental group were collected for further inspection. For immunostaining, cryosections were air-dried for about 1 hours at room temperature, following by washing in PBS for three times and incubation with blocking buffer (5% Goat serum) for 30 minutes at room temperature and then incubated with primary antibody overnight at 4°C. Signals were detected by using Alexa fluorescence-conjugated secondary antibodies (Invitrogen) for 1 h at room temperature. Images were acquired using a laser scanning confocal microscope (Leica, Germany). Quantitative analyses were performed on more than three slices using Image J software. For details, images were merged using the Image color-merge channels function; Z-projects and color-merge channels function was used to merge images. In the stack, we use an orthogonal view to obtain the signals on the XZ or YZ axis. Merged signals and split channels were used to delineate the signals at single-cell resolution. For quantification of the percentage of RGS5 ^high^ FN^+^ cells, the number of double positive cell is set as numerator while the number of DAPY ^+^ cells is set as denominator. This result as ratio is then transformed into percentage. Aortic roots were dissected and snap frozen in liquid nitrogen after embedded with OCT. Serial frozen sections were cut at 8 μm thickness and stained with Oil Red O using standard procedure.

### Flow cytometry analysis

Cells from different regions of aorta were isolated and blocked with anti-CD16/32 antibody for 30 min on ice. Surface markers were then stained for 30 min on ice. And then cells were fixed and permeabilized by Intracellular Fixation & Permeablization buffer set (BD), followed by intracellular antigen staining. Fluorescence labeled cells analyzed was performed using guava EasyCyte system (Merck), and FlowJo V10 software was used for data analysis and quantification.

### Aorta isolation and cell dissociation

The aorta was dissected from the root (distal to the aortic valve) to the femoral artery bifurcation. The isolated aorta included aortic arch, ascending, descending, thoracic, and abdominal portions. Perivascular fat was dissected from the vascular tissue prior to dissociation and single cell analysis. To generate single-cell suspensions, endothelial cells were removed manually from dissected aortas using a cotton bud and vessels incubated for 10 min in Collagenase Type IV (1 mg/ml, sigma) and porcine pancreatic elastase (1 U/ml, Worthington) in DMEM to allow for separation of the adventitia and medial cell layers. Tissue isolated from 4 animals was pooled and preparation of a single cell suspension of aortic cells was performed using an enzymatic digestion protocol. Briefly, the isolated medial cell layers of whole aorta was finely cut and first digested by 1X Aortic Dissociation Enzyme Solution (1.0 mg/mL collagenase type I, 1.0 mg/mL collagenase type I, 0.20 mg/mL collagenase type XI, 1.8U/mL porcine pancreatic elastase, 2.0 mM CaCl_2_) for 20 min at 37°C, followed by digestion with a mixture of 1X Aortic Dissociation Enzyme Solution and 10 U/mL DNAase Ⅰ for 20 min at 37°C. The cell suspension was strained through a 40 μm filter, treated with ACK lysis buffer for 5 minutes at room temperature for red blood cell lysis, and washed twice with PBS.

### Western blot analysis

Equal amounts of extracts (30 μg) were separated by 10% SDS-PAGE and transferred to PVDF membrane. After blocking with 5% milk in TBST, the membranes were incubated with primary antibodies against RGS5 (1:1000, Proteintech Group), β-actin (1:1000, Cell Signaling Technology) at 4°C overnight. After incubating with HRP-conjugated secondary antibody (1: 20 000, Abcam), the blots were visualized using GE ImageQuant™ LAS 4000 detection system. Band intensities were quantified with Image Pro Plus 6.0 software.

### RNA isolation and quantitative reverse transcription-PCR (RT-qPCR)

Total RNAs of tissue samples were isolated using TRIzol reagent (Life Technologies). To quantify the amount of mRNA, cDNA was synthesized using the M-MLV First Strand Kit (Life Technologies), and quantitative PCR was performed using SYBR Green qPCR SuperMix-UDG (Life Technologies). Relative mRNA expression was normalized to GAPDH levels, using the 2^−△△Ct^ method. The average threshold cycle for each gene was determined from at least three independent experiments.

### ScRNA-seq library preparation and sequencing

Single cell suspensions (300–500 living cells per micro liter) were loaded on a Chromium Single Cell Controller (10x Genomics) to generate single-cell Gel Bead-In-Emulsions (GEM) by using single cell 3 ’Library and Gel Bead Kit V3 (10x Genomics, 1000075) and Chromium Single Cell B Chip Kit (10x Genomics, 1000074). In short, single cells were suspended in PBS containing 0.04% BSA. About 6,000 cells were added to each channel, and the target cell will be recovered was estimated to be about 3,000 cells. Captured cells were lysed and the released RNA was bar-coded through reverse transcription in individual GEMs. Reverse transcription was performed on a S1000TM Touch Thermal Cycler (Bio Rad) at 53°C for 45 min, followed by 85°C for 5 min, and hold at 4°C. The cDNA was generated and then amplified, and quality assessed using an Agilent 4200 (performed by CapitalBio Technology, Beijing). Single-cell RNA sequencing libraries were constructed using Single Cell 3’Library and Gel Bead Kit V3. The libraries were finally sequenced using an Illumina Novaseq6000 sequencer with a sequencing depth of at least 100,000 reads per cell with pair-end 150 bp (PE150) reading strategy (performed by CapitalBio Technology, Beijing).

### Processing raw data from scRNA-seq

First, FASTQ files were processed by the function “cell ranger count” of software Cell Ranger (10× Genomics, Version 3.0.2) to generate feature counts of every single cell. The reference genome was the mouse mm10 genome (10× Genomics, Version 3.0.0.). Gene-expression matrix was then obtained by demultiplexing, barcode processing, and single-cell 3’gene counting. The gene expression matrix was imported to the Seurat (version 3.0) R package for quality control and downstream analysis. The gene expression matrix obtained by Cell Ranger was employed to format a Seurat object. For quality control, genes with <200 cells detected were filtered out and cells with >500 and <10000 genes and a mitochondrial gene percentage of <30% were retained.

### Cell clustering of scRNA-seq data

The filtered gene expression data of counts were analyzed by the Seurat (Version 3.0) package. Global scaling normalization was applied with the “Normalize Data” function via the “Log Normalize” method and a scale factor 100,000 for modified STRT-seq and 10,000 for 10×scRNA-seq respectively according to their number of transcriptional counts. Highly variable genes were identified by “Find Variable Genes” and used for principal component analysis. Significant principal components that were enriched with low p value genes were used for cell clustering. The resolution of “Find Clusters” was set to 0.5 for modified STRT-seq and 0.1 for 10×scRNA-seq respectively because the resulting clusters were consistent with the mapping of known markers.

### Identification of differentially expressed genes (DEGs) and biomarkers

DEGs from scRNA-seq were identified by “Find All Markers” or “Find Markers” using the “Wilcox” test, which returned “*p*_val_adj” using the Bonferroni correction and the log-transformed fold change, “avg_logFC”. Biomarkers of different clusters were selected from DEGs with *p*_val_adj ≤ 0.05 and avg_logFC ≥ 1.0.

### Enrichment analysis

GO enrichment, KEGG enrichment and Reactome enrichment of cluster markers were performed using KOBAS software with Benjamini-Hochberg multiple testing adjustment, using top 20 markers gene of cluster. The results were visualized using R package.

### Construct cell trajectories

Single-cell trajectories were built with Monocle (R package) that introduced pseudotime. Genes were filtered by the following criteria: Expressed in more than 10 cells; the average expression value was greater than 0.1; Qval was less than 0.01 in different analysis. DEGs were identified by the “Find Markers” function of the Seurat packages were set as “ordering_genes”. The DDR Tree algorithm was used for dimensionality reduction. Modules of DEGs that covaried along the pseudotime were demonstrated by “plot_pseudotime_heatmap”.

### Statistical analysis

Data are presented as the means ± SEM. p value was calculated using GraphPad Prism software by two-tailed Student’s t tests. For multiple comparisons, two-way ANOVA or one-way ANOVA, followed by the recommended Holm-Sidak method, was used. Alternatively, statistical methods were included in figure legends.

## Results

### Overview of the VSMC subpopulation composition in the healthy aorta of mice

**Fig 1:**
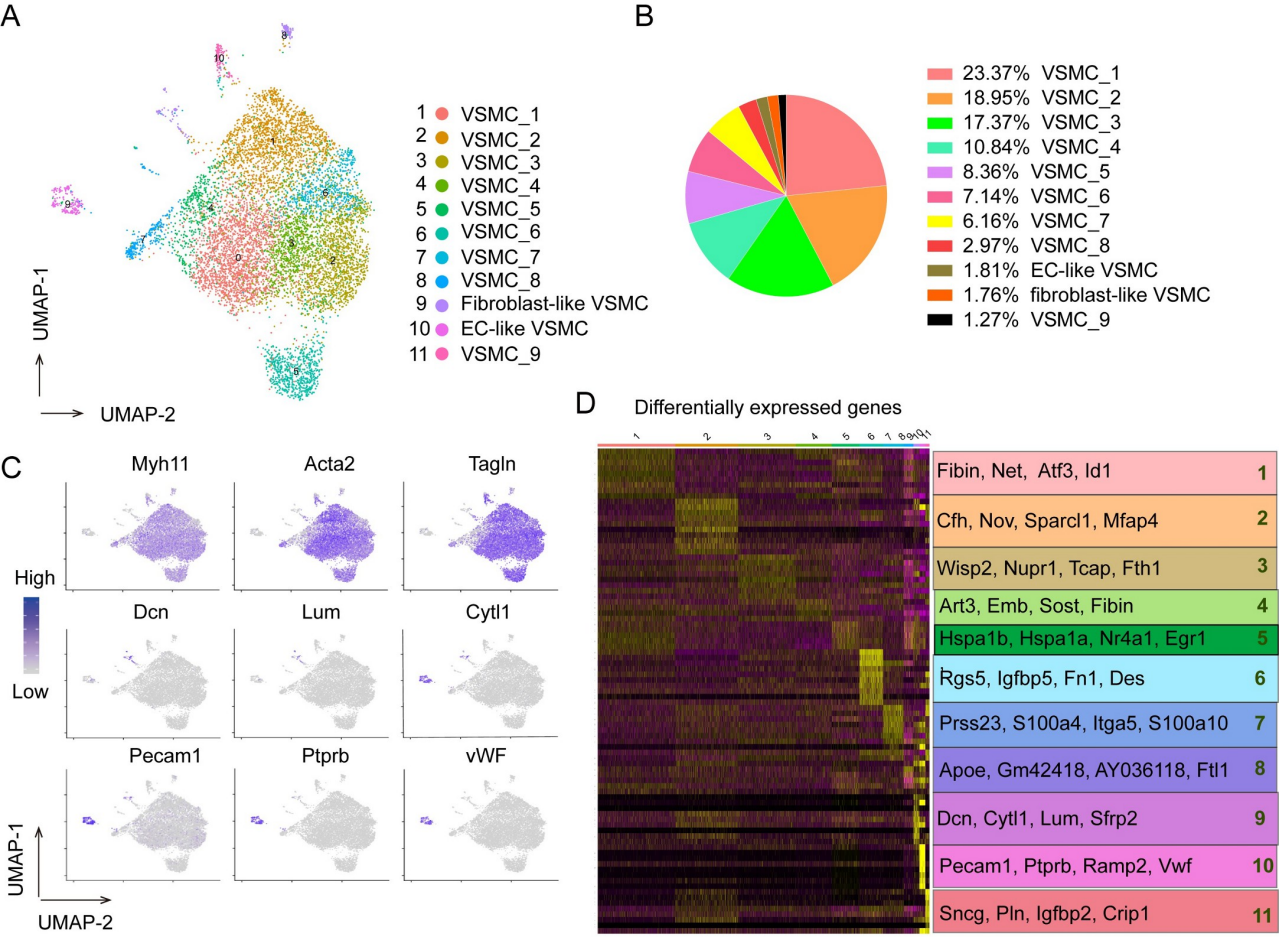
Overview of the composition of VSMC subpopulations in healthy mouse aorta. (A) A UMAP plot showing *Myh11*-expression cells colored according to cluster. EC indicates endothelial cells. (B) The percentage of each subpopulation of *Myh11*-expression cells. (C) Relative expression of several marker genes in *Myh11*-expression cells. Cells were projected onto a UMAP plot. (D) Heat map (left) showing the top 10 differentially expressed gene in each cluster. The top 4 marker genes were shown to the right.

To determine the subpopulation structure and molecular characteristics of VSMCs in healthy artery, we initially performed scRNA-seq on the medial layer of the normal mouse aortas sampled from four healthy mice. Enzymatic dissociation protocol was used to prepare single cells and produced high quality single cells (S1 Fig). Single-cell capture and cDNA library preparation were performed using the 10X Genomics Chromium platform followed by library preparation, multiplexed sequencing, quality control (S2 Fig), and analysis using the Seurat scRNA-seq analysis package in R (Methods). We obtained 10138 high-quality single cells, and detected an average number of 3800 genes per cell. To enable transcriptomic analysis across the VSMCs spectrum, we isolated the *Myh11*-expressing cells from the dataset, which segregated into eleven clusters, including nine canonical VSMC subpopulations (VSMC_1-VSMC_9), one EC-like VSMC cluster and one fibroblast-like VSMC cluster, on the basis of their respective molecular features (Fig 1A–1C). In order to determine whether separate clusters identified within *Myh11*-expressing cells represent discrete VSMC subpopulations or a continuous phenotypic gradient, the markers for each subpopulation were plotted via heatmap (Fig 1D). VSMC_6, VSMC_7, EC-like VSMC cluster and fibroblast-like VSMC cluster express distinct and non-overlapping markers, suggesting the presence of discrete subpopulations. Conversely, the other subpopulations expressed overlapping markers, suggesting the presence of a continuous phenotypic gradient rather than true subpopulations. Notably, VSMC_6 subpopulation present better independent and comprise the largest population of the four discrete subpopulations in our analysis (Fig 1A and 1B).

### Temporal characterization of VSMC subpopulations

**Fig 2:**
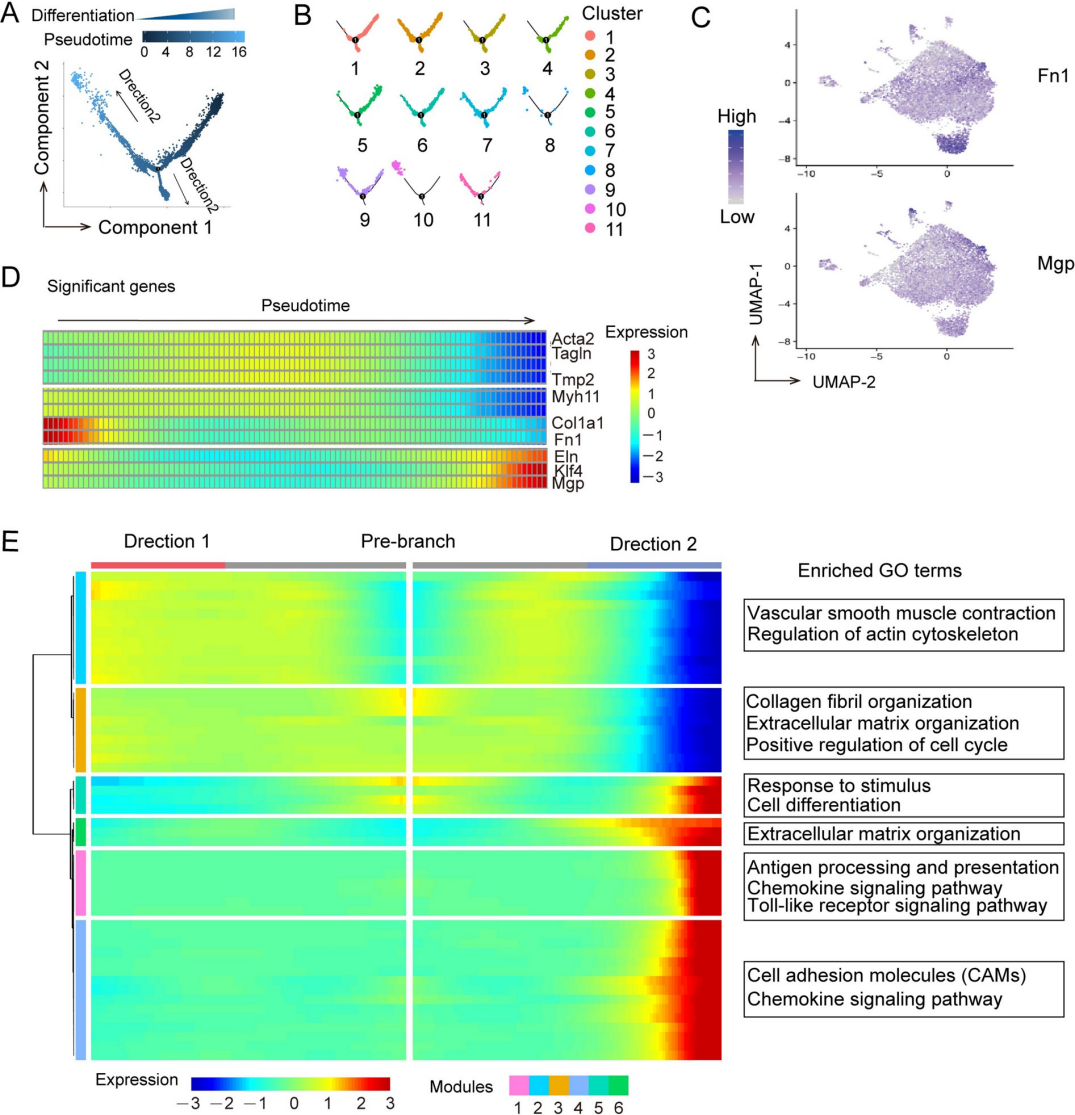
Pseudotime analysis of the medial VSMC subpopulations. (A) Pseudotime trajectory of *Myh11*-expression cells. (B) Cells ordering from different cluster along the pseudotime trajectory. (C) Relative expression of *Fn1* and *Mgp* in *Myh11*-expression cells. Cells were projected onto a UMAP plot. (D) Heat map of the selected significantly changed genes along the pseudotime trajectory. (E) Branched heat map of the relative expression of 50 genes along the inferred pseudo time axis (cataloged hierarchically into six gene modules) corresponding to Direction 1 and Direction 2, and representative GO terms enriched in each gene module.

Pseudotime trajectory analysis was used to inspect the differentiation state of each VSMC subpopulation, which maps a trajectory along a continuous cell state transition and then determines each cell’s progress down this trajectory based upon cell’s transcriptome. We found one main path, with two major branches (Fig 2A). Cells at the same part derived from several VSMC subpopulations, and cells from the same subpopulation located at different parts of the trajectory (Fig 2B), suggesting that the differentiation degree of VSMCs in each subpopulations was not uniform within the health artery. Although it has known that VSMCs display a fully differentiated state in health vessel, our scRNA-seq data showed that some VSMCs in normal artery expressed low levels of contraction-related genes such as *Tagln* and *Acta2* (Fig 1C), and highly expressed ECM components such as *Fn1* [16] and *Mgp* [6], known as dedifferentiation VSMC marker genes (Fig 2C). Heatmap of significantly changed genes along the pseudotime trajectory showed that the expression of differentiation markers (*Acta2*, *Tagln*, *Tmp2* and *Myh11*) were first increased and then decreased; meanwhile dedifferentiation VSMC markers (*Fn1*, *Eln* and *Mgp*) and regulator *Klf4* [13] displayed the reverse change trend along pseudotime trajectory (Fig 2D), indicating that there were lower differentiated cells in each VSMC subpopulations. Notably, these lower differentiated VSMCs were different from synthetic cells that highly expressed *Spp1*, *MMPs*, *Icam-1* and *Lum* [16].

Additionally, to further define the gene expression characteristics and the potential cellular functions of VSMC subpopulations along pseudotime, we performed branched expression analysis modeling (BEAM), followed by hierarchical clustering analysis, allowing us to identify six different gene expression modules (modules 1–6) (Fig 2E). GO enrichment analyses showed that pre-branch cells high expressed ECM organization, and cells of “direction 1” branch mainly involved in VSMCs contraction and actin cytoskeleton construction, whereas cells of “direction 2” branch highly expressed inflammatory activation, chemotaxis and adhesion genes (Fig 2E), inferring that there was significant development heterogeneity within each VSMC subpopulation.

### Functional diversity of VSMC subpopulations

**Fig 3:**
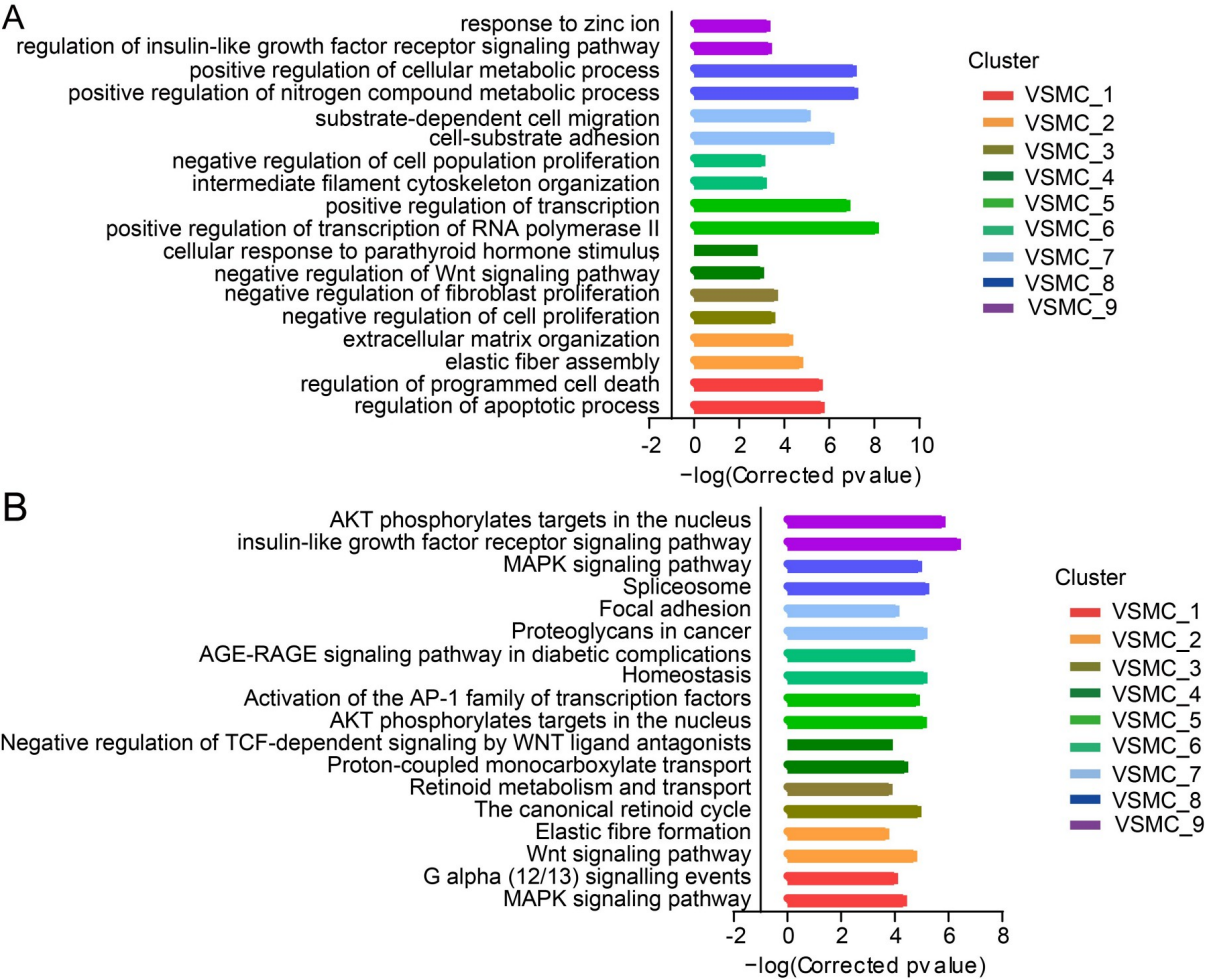
Functional diversity of VSMC subpopulations. (A) Gene set enrichment analyses showing top enriched biological processes in each VSMC subpopulation. (B) Reactome or KEGG pathway enrichment analyses of VSMC subpopulations.

The Gene Ontology (GO) analysis of differentially expressed genes (DEGs) in each cluster revealed functional diversity of VSMC subpopulations (Fig 3A). VSMC_1 subpopulation displayed functional enrichment in biological behaviors related to apoptosis, and VSMC_2 was associated with elastic fiber and extracellular matrix (ECM) assembly. Proliferation was suppressed in VSMC_3 and VSMC_4, and cells in VSMC_5 highly expressed the stress response genes such as *Hspa1b*, *Hspa1a*, *Nr4a1*, *Fosb* and *Egr1*, suggesting activation of the stress response in this subpopulation [17] (Fig 1D). Furthermore, genes related to inhibition of cell proliferation and cytoskeletal construction were activated in cells of VSMC_6. Genes in VSMC_7 exhibited enrichment in proliferation, migration and adhesion, as *Vcam1*, *FN1*, *Mgp*, *S100a4* and *S100a10* were highly expressed in this subpopulation (S7 File), indicating that these cells may undergo phenotypic switching. Meanwhile, *Lgals3* was differently expressed in this subpopulation (S7 File), inferring that macrophage-like VSMCs [18, 19] existed in normal media. Besides, VSMC_8 subpopulation highly expressed genes associated with cellular metabolic process.

In order to further identify the functions of VSMC subpopulation, pathway enrichment was performed on the top 1000 markers of each subpopulation against the Reactome [20] or KEGG pathway database (Fig 3B). MAPK and G protein signaling were activated in VSMC_1 subpopulation. Wnt signaling involved in extracellular matrix remodeling in VSMC_2 subpopulation. Retinoid signaling mediated the proliferation inhibition of VSMC_3 subpopulation. VSMC_4 subpopulation was characterized by proton-coupled monocarboxylate transport and negative regulation of ternary complex factor (TCF)-dependent signaling by Wnt ligand antagonists. VSMC_6 subpopulation uniquely expressed homeostasis and advanced glycation end-product (AGE)-(advanced glycation end-product receptor) RAGE signaling pathway related genes, inferring that this subpopulation plays a key role in maintaining vascular homeostasis and vascular diseases. VSMC_7 was distinct based on the activation of proteoglycans and focal adhesion signaling. In VSMC_8 subpopulation, spliceosome signaling pathway was activated to maintain the active metabolism. Additionally, insulin-like growth factor receptor signaling pathway was activated in VSMC_9 subpopulation. The molecular differences between VSMC subpopulations underlie distinct functions, suggesting that VSMCs are more heterogeneous than previously recognized.

### Spatial distribution characterization of RGS5^high^ VSMC subpopulation

**Fig 4:**
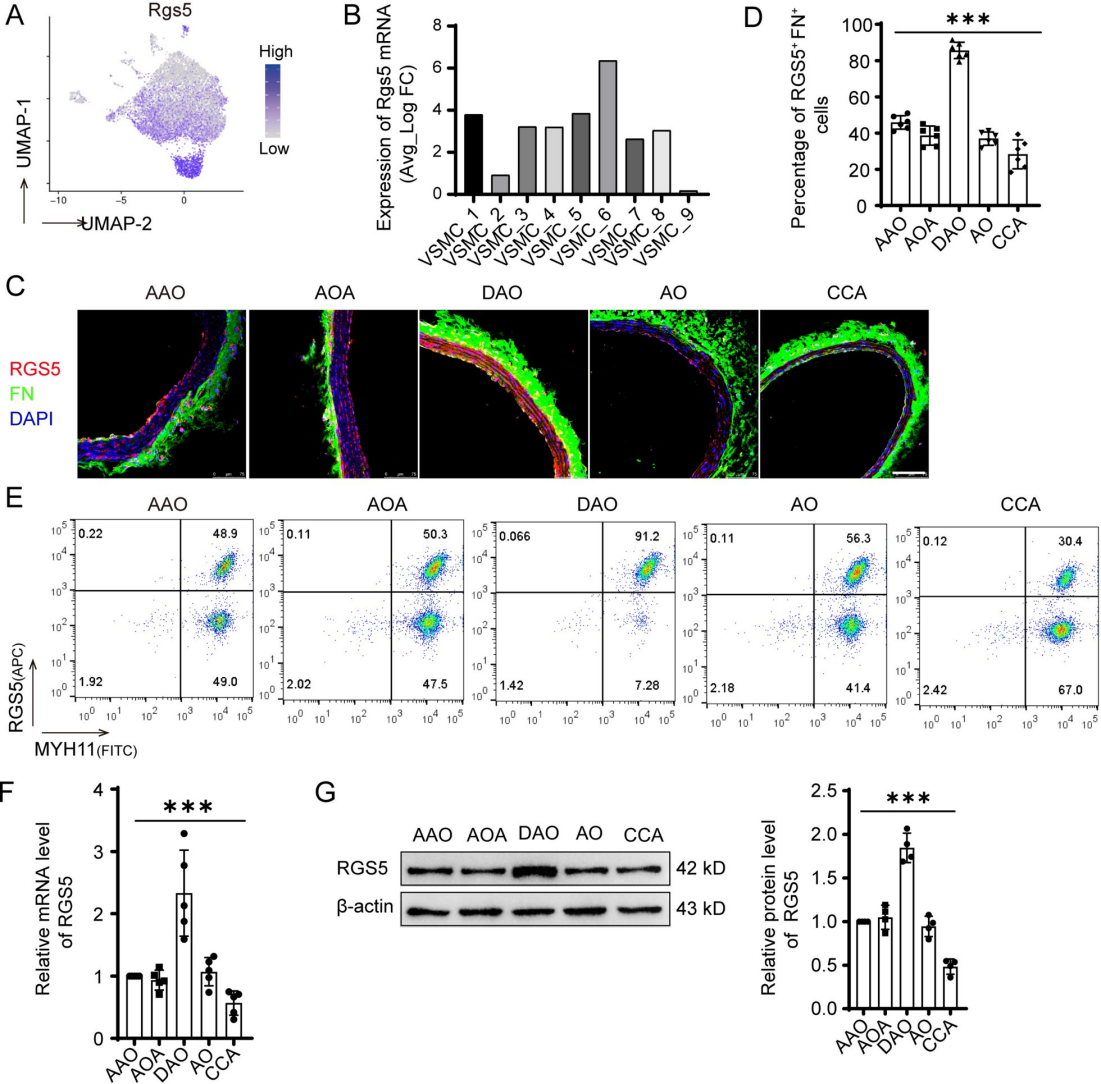
Distribution characterization of RGS5^high^ VSMC subpopulation. Spatial (A) Relative expression of RGS5 in *Myh11*-expression cells. Cells were projected onto a UMAP plot. (B) Expression level of *Rgs5* mRNA in each VSMC subpopulation according to the single cell RNA sequencing data. (C)Immunofluorescence microscopy images for RGS5 and FN in different aorta regions. AAO indicates the ascending aorta; AOA, aortic arch; DAO, descending thoracic aorta; AO, abdominal aorta; CCA, common carotid artery. Scale bur = 75μm. (D) The percentage of RGS5^+^ and FN^+^ VSMCs in different aorta regions. Individual replicates and their means are indicated and error bars show s.e.m, n = 6. ***P<0.0001. (E) The percentage of RGS5^+^ and MYH11^+^ cells in different aorta regions analyzed by flow cytometry in different aorta regions. (F) RT-qPCR for the expression of RGS5 in different aorta regions. n = 5, ***P<0.0001. (G) Western blot for RGS5 expression in different aorta regions. n = 5, ***P<0.0001.

As VSMC_6 subpopulation clustered separately in Uniform Manifold Approximation and Projection (UMAP) plot and might have a critical role in vascular homeostasis, next we intended to inspect the spatial location of this subpopulation *in situ*. RGS5 is a member of regulator of G protein signaling molecules family, which has been reported to be a marker of VSMCs and pericytes [21]. Interestingly, RGS5 is specific highly expressed in VSMC_6 (fold change = 2.11) accompanied with high expression of *FN1* (fold change = 1.48) (Figs 4A and 1D and S2 File), and however, the expression of other pericyte markers NG2, PDGFR-β, Cd146, Nestin were not higher in this subpopulation (S3 Fig). We then used these genes as markers and named this subpopulation as RGS5^high^ VSMCs. We performed duplex chromogenic in immunofluorescence for RGS5 (red) and Fibronectin (FN) (green) on the common carotid artery (CCA) and different regions of the aorta, including the ascending aorta (AAO), the aortic arch (AOA), the descending thoracic aorta (DAO) and the abdominal aorta (AO). Results showed that RGS5 and FN double-positive cells were mainly distributed in the descending thoracic aorta, and fewer in other regions of aorta and common carotid artery (AAO, 45.88±1.52% vs. AOA, 38.67±2.16% vs. DAO, 85.61±1.83% vs. AO, 36.99±1.51% vs. CCA, 28.34±3.27%) (Fig 4B and 4C). The difference was further confirmed by flow cytometry. The number of RGS5 and myosin heavy chain 11 (MYH11) double positive cells was higher in DAO than the other regions (Fig 4D). Furthermore, the higher RGS5 transcript and protein levels in DAO were verified by RT-qPCR, and Western blotting (Fig 4E and 4F). As enrichment analysis of the highly expressed genes in the RGS5^high^ VSMC subpopulation showed that this subpopulation was mainly involved in vascular homeostasis (Fig 3A), we speculated that the distribution of RGS5^high^ VSMC subpopulation may be associated with the resistance of the descending thoracic aorta to diseases, as observed in atherosclerosis [22].

### The reduction of RGS5^high^ VSMC subpopulation during vascular disease

**Fig 5:**
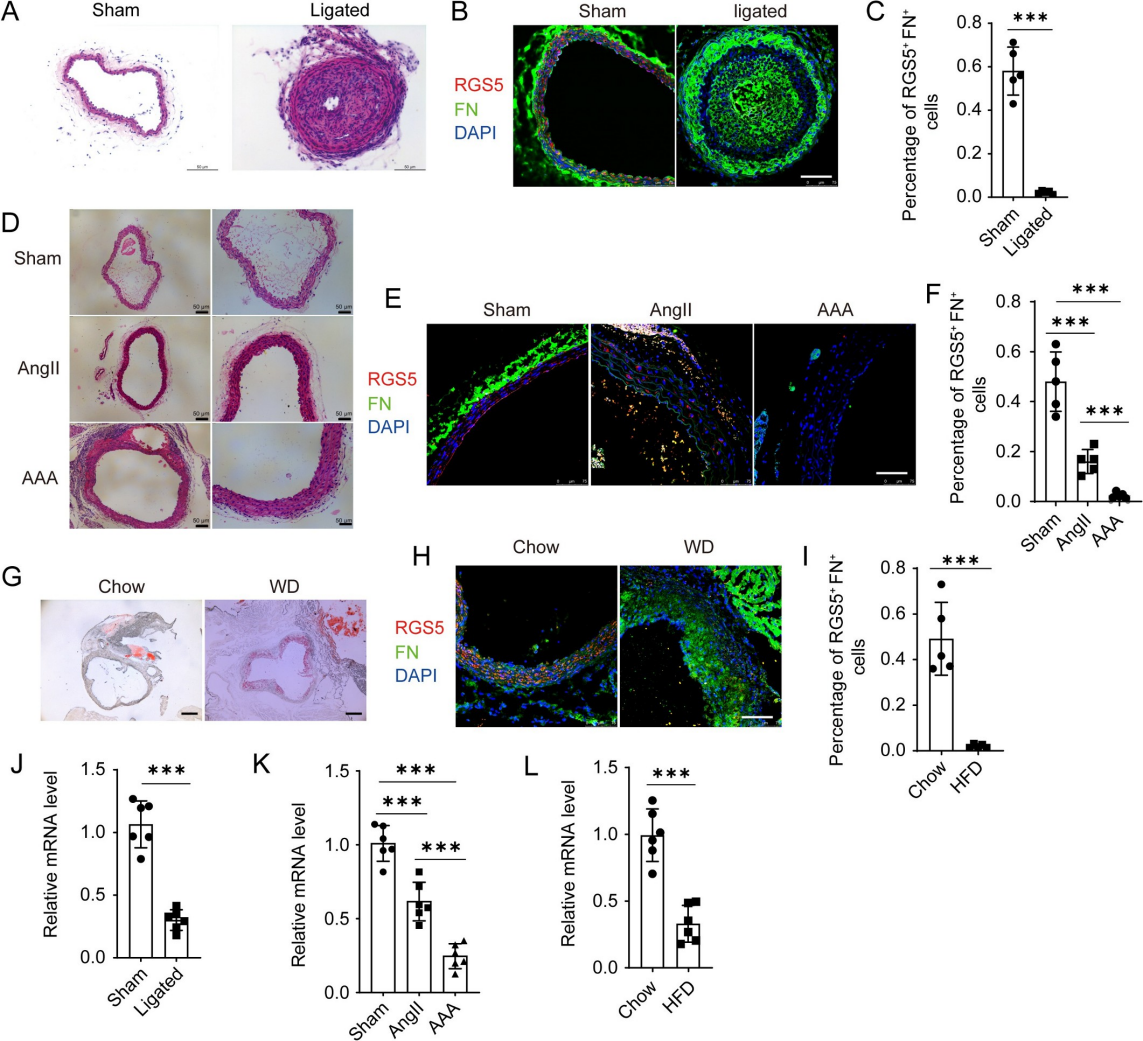
The role of RGS5^high^ VSMC subpopulation during vascular remodeling. (A) Representative HE staining images of neointima. Scale bar = 50μm. (B) Representative immunofluorescence microscopy images for RGS5 and FN in neointima. Scale bar = 75μm. (C) The percentage of RGS5 ^high^ and FN^+^ VSMCs in neointima. Individual replicates and their mean are indicated and error bars show s.e.m, n = 6. ***P<0.0001. (D) Representative HE staining images of AngII treated abdominal aorta of mice. Scale bar = 50μm. (E) Representative immunofluorescence microscopy images for RGS5 and FN in AngII treated abdominal aorta. Scale bar = 75μm. (F) The percentage of RGS5 ^high^ and FN^+^ VSMCs in AngII treated abdominal aorta. Individual replicates and their mean are indicated and error bars show s.e.m, n = 5. ***P<0.0001. (G) Representative images of Oil Red O staining for aortic roots sections. Scale bar = 50μm. (H) Representative immunofluorescence microscopy images for RGS5 and FN in atherosclerotic plaque of aortic roots sections. Scale bar = 75μm. (I) The percentage of RGS5 ^high^ and FN^+^ VSMCs in atherosclerotic plaque. Individual replicates and their mean are indicated and error bars show s.e.m, n = 5. ***P<0.0001. (J-L) RT-qPCR analysis for the expression of RGS5 in ligated common carotid artery, AngII treated abdominal aorta or aorta with plaque. n = 6. ***P<0.0001.

In order to further confirm the functional identity of RGS5^high^ VSMCs, several animal models of vascular disease were performed, including carotid artery ligation, abdominal aortic aneurysm and atherosclerosis. First, we prepared carotid artery ligation model for cryosections, and results showed that the proportion of RGS5^high^ VSMCs was decreased in neointima compared to the normal carotid artery (Sham, 58.05±4.91% vs. Ligated, 2.2±0.34%) (Fig 5A–5C). Similarly, the number of RGS5 and Fn positive cells was significantly reduced in the aortas of mice infused with angiotensin II (AngII), and it was nearly disappeared with aneurysm formation (Sham, 48.02±5.32% vs. AngII, 16.06±2.15% vs. AAA, 2.44±0.54%) (Fig 5D–5F). To determine whether similar change occurs in atherosclerosis, we established mouse models of atherosclerosis using density lipoprotein receptor deficient (*Ldlr*^*−/−*^) mice fed on Western diet (WD) for 12 weeks, and analyzed the proportion of RGS5^high^ VSMCs in plaque. We observed the consistent results that RGS5 and Fn positive cells declined considerably in atherosclerotic plaque (Chow, 49.14±7.14% vs. WD, 2.04±0.38%) (Fig 5G–5I). Additionally, the levels of RGS5 were markedly repressed in vascular lesions, as shown by RT-qPCR analysis (Fig 5J–5L). Based on these findings, it is likely that RGS5^high^ VSMCs may play critical role in maintaining vascular homeostasis, and loss of this subpopulation could exacerbate vascular diseases.

## Discussion

Although accumulating evidence suggests that there is large heterogeneity in VSMCs, our understanding of VSMCs-driven disorders is still hampered by our limited knowledge of inter-subpopulation differences and subpopulation-specific functions in healthy artery. In the present study, we identified the detailed transcriptome characteristics and functions of VSMC subpopulations in healthy artery. Pseudotime bioinformatics analyses revealed that differentiation degree and development were heterogeneous within each VSMC subpopulation. Importantly, we discovered a homeostasis-relevant VSMC subpopulation with higher expression of RGS5 and FN, which was enriched in the descending thoracic aorta and markedly reduced in vascular disease, suggesting that this subpopulation may affect the resistance or susceptibility of specific vascular-bed to disease. Thus, our study made a key step towards comprehensively view VSMCs function in homeostasis and diseases.

G protein–coupled receptors (GPCRs) detect a wide array of extracellular molecules, activate intracellular signaling, and lead to multiple cellular responses [23]. RGS, as a negative regulator of GPCR signaling, is the most highly and differently expressed RGS-R4 subfamily member in arterial smooth muscle. RGS5 is critical to vascular stability in newly formed vascular beds [24] and to blood pressure regulation [24, 25]. In adult mice, the expression of RGS5 presents vascular-bed specific, higher in the descending aorta and renal arteries [26, 27] and it was differentially expressed between aortic arch and the descending aorta [1]. Previous studies have demonstrated that RGS5 is involved in vascular remodeling in tumors [28], hypertension [29, 30] and atherosclerosis [31, 32] via regulation of cellular stress responses, proliferation and constriction, inflammatory response, and vascular barrier function. The expression of RGS5 is repressed by PDGF and known to control vascular contractility by accelerating the inactivation of Gα-dependent signaling, suggesting that that RGS5 expression is a critical mediator of both VSMC contraction and potentially, arterial remodeling [33, 34]. These findings establish a significant, distinct, and causal role of RGS5 in vascular homeostasis and remodeling. Recently, single-VSMC transcriptomes revealed that RGS5 is one of the top variably expressed genes between the aortic arch and descending thoracic aorta [1], inferring that RGS5 keeps the balance right between vascular physiology and pathology. Consistently, we demonstrated that the vascular-bed different location of RGS5 marked VSMC subpopulation resulted in the vascular-bed different expression of RGS5. The current study is also in accordance with previous study regarding vascular bed-specific differences in RGS5 expression [26], which revealed that origin-dependent epigenetic program regulates vascular bed-dependent regulation of *RGS5* transcription. On one hand, our study validates the previous finding in single cell resolution; on the other hand, it is inspiring for us to combine scRNA-seq and epigenetics for molecular mechanism investigation. Moreover, the proportion of RGS5^high^ VSMCs was significantly decreased in the vascular tissues underlying neointima hyperplasia, aortic aneurysm formation and atherosclerosis in mice, suggesting that RGS5^high^ VSMC subpopulation plays a critical role in maintaining vascular homeostasis and may act as safeguard in vascular remodeling. Of note, RGS5 prevents VSMC proliferation during neointimal lesion formation in vivo [35], suggesting that RGS5 has a key role in maintaining the VSMC contractile phenotype in molecular level. However, the mechanism of RGS5^high^ VSMCs in maintaining vascular homeostasis remains to be futher investigated in cell level.

While our study has provided a comprehensive view of VSMCs in healthy and injured artery, we acknowledged the limitations of our study. Firstly, we need to utilize dual inducible genetic tracing systems and/or manipulate the cell number of RGS5^high^ subpopulation in vivo to further confirm the function of RGS5^high^ VSMCs in vascular homeostasis. Secondly, it is important for exploring the signaling pathway and the mechanism underlying RGS5 regulating the pheontypic switching of VSMCs and vascular remodeling. Additionally, the findings and translational value of our study need to be further investigated using human tissues.

Overall, our study not only provides a wealth of reference information on VSMC subpopulations in healthy artery, but also offers primary insights into the role of RGS5^high^ VSMC subpopulation in vivo—knowledge that could potentially serve a therapeutic purpose.

## Supporting information

S1 Fig Quality identification of single cell suspension. (A) Immunofluorescence staining of living and dead cells. FL1, living cells; FL2, dead cells; BL, bright field. (B) Histogram of fluorescence distribution for living and dead cells. C, Histogram of cell diameter distribution.
(TIF)

S2 Fig Quality control of single cell RNA sequencing. (A) Average error rate distribution of base pairing. (B) Base content distribution. (C) Effective cell count analysis (D) Sequencing saturation curve. (E) Violin diagram for gene number and UMI numbers. (F) Correlation of gene number and UMI number.
(TIF)

S3 Fig RT-qPCR analysis for the expression of other pericyte marker genes in each VSMC subpopulation. (TIF)

S1 File The top differentially expressed genes of VSMC_1 subpopulation. (PDF)

S2 File The top differentially expressed genes of VSMC_2 subpopulation. (PDF)

S3 File The top differentially expressed genes of VSMC_3 subpopulation. (PDF)

S4 File The top differentially expressed genes of VSMC_4 subpopulation. (PDF)

S5 File The top differentially expressed genes of VSMC_5 subpopulation. (PDF)

S6 File The top differentially expressed genes of VSMC_6 subpopulation. (PDF)

S7 File The top differentially expressed genes of VSMC_7 subpopulation. (PDF)

S8 File The top differentially expressed genes of VSMC_8 subpopulation. (PDF)

S9 File The top differentially expressed genes of VSMC_9 subpopulation. (PDF)

S1 Raw data Raw blot image for Fig 4G. (TIF)
