# Supplementary figures and images for: A regulator of G protein signaling 5 marked subpopulation of vascular smooth muscle cells is lost during vascular disease
Source: PLoS One. 2022 Mar 23;17(3):e0265132. doi: 10.1371/journal.pone.0265132 (PMC8942229; doi:10.1371/journal.pone.0265132)

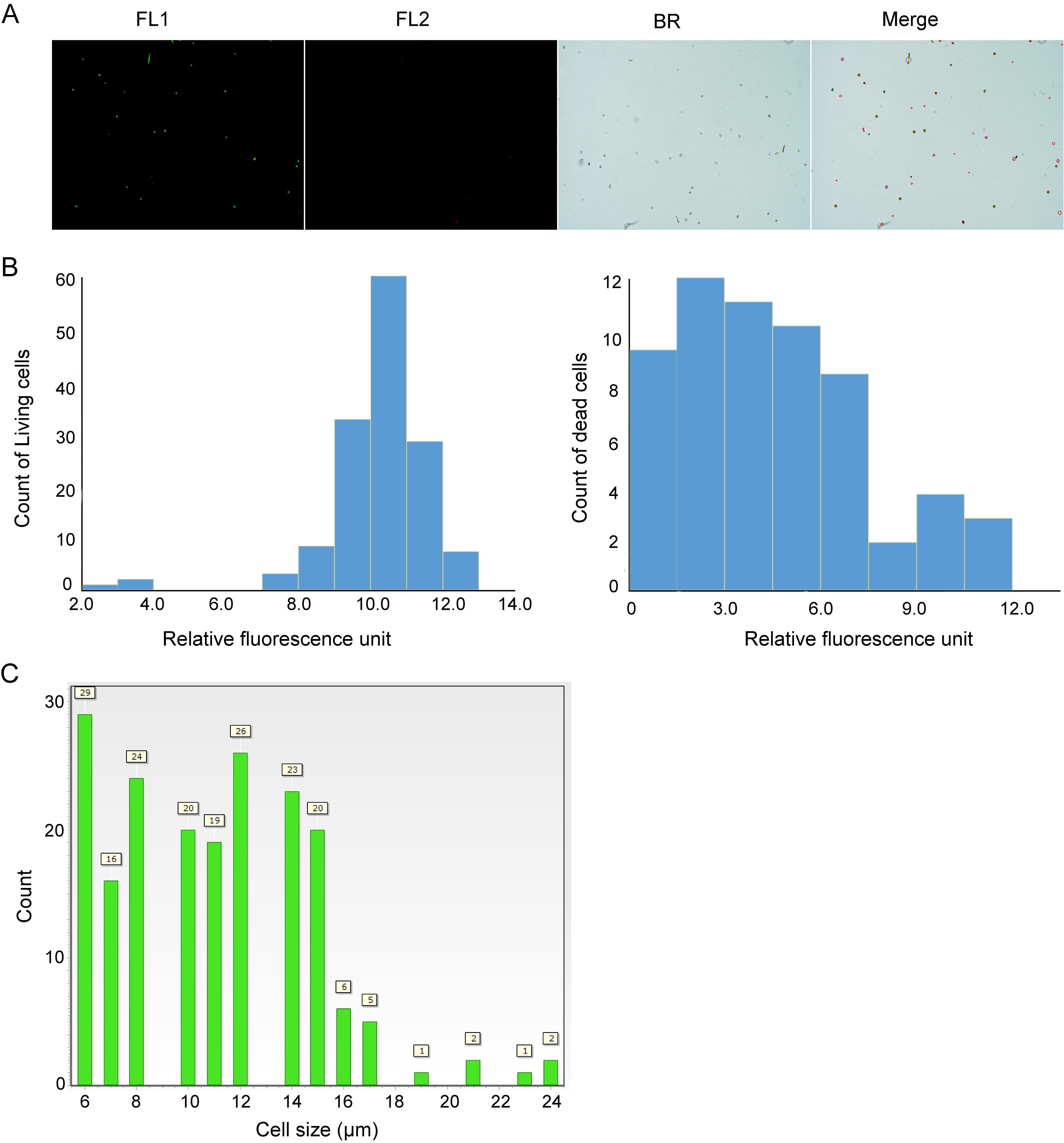

Supplement: S1 Fig — (A) Immunofluorescence staining of living and dead cells. FL1, living cells; FL2, dead cells; BL, bright field. (B) Histogram of fluorescence distribution for living and dead cells. C, Histogram of cell diameter distribution. (TIF) [file pone.0265132.s001.tif]

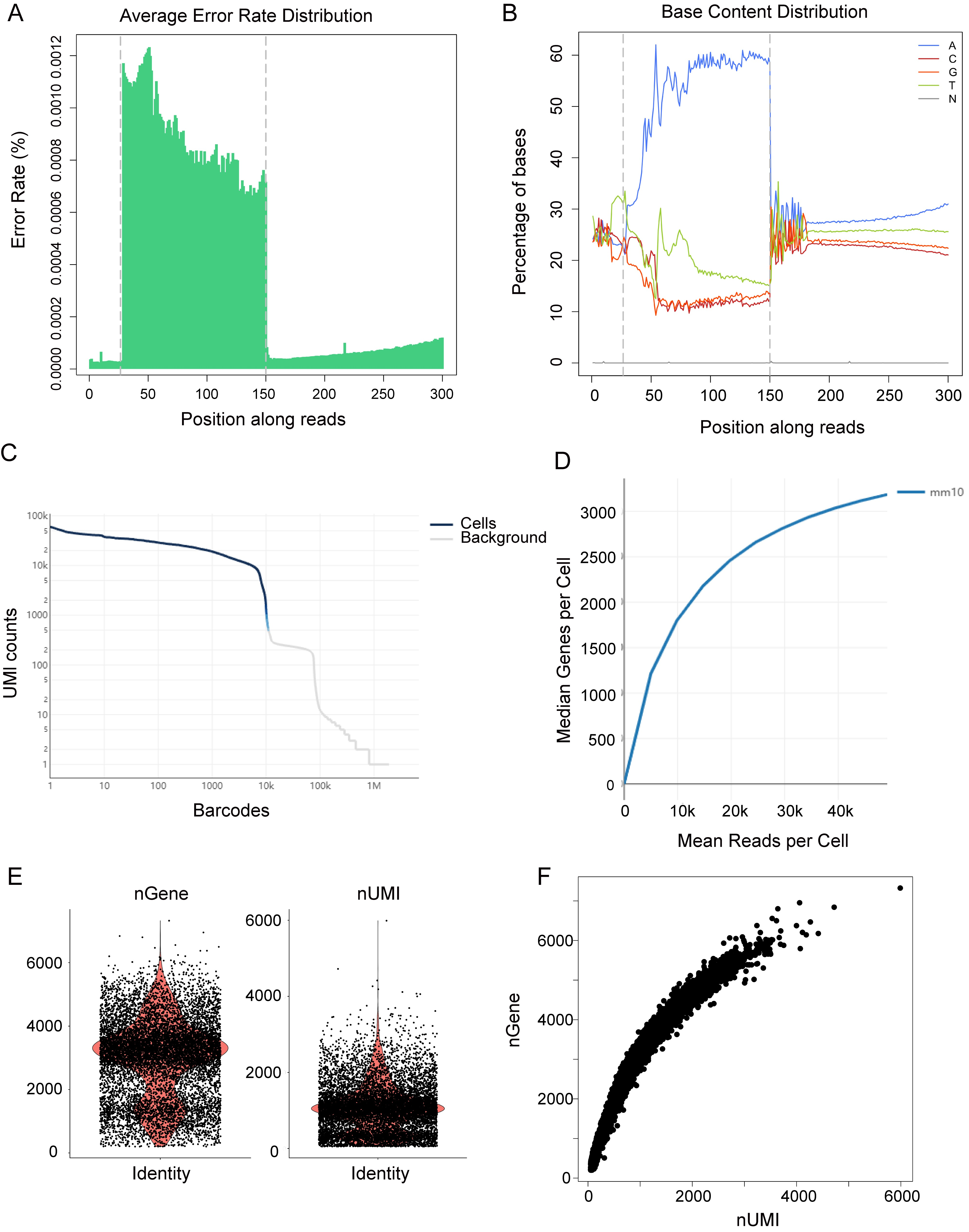

Supplement: S2 Fig — (A) Average error rate distribution of base pairing. (B) Base content distribution. (C) Effective cell count analysis (D) Sequencing saturation curve. (E) Violin diagram for gene number and UMI numbers. (F) Correlation of gene number and UMI number. (TIF) [file pone.0265132.s002.tif]

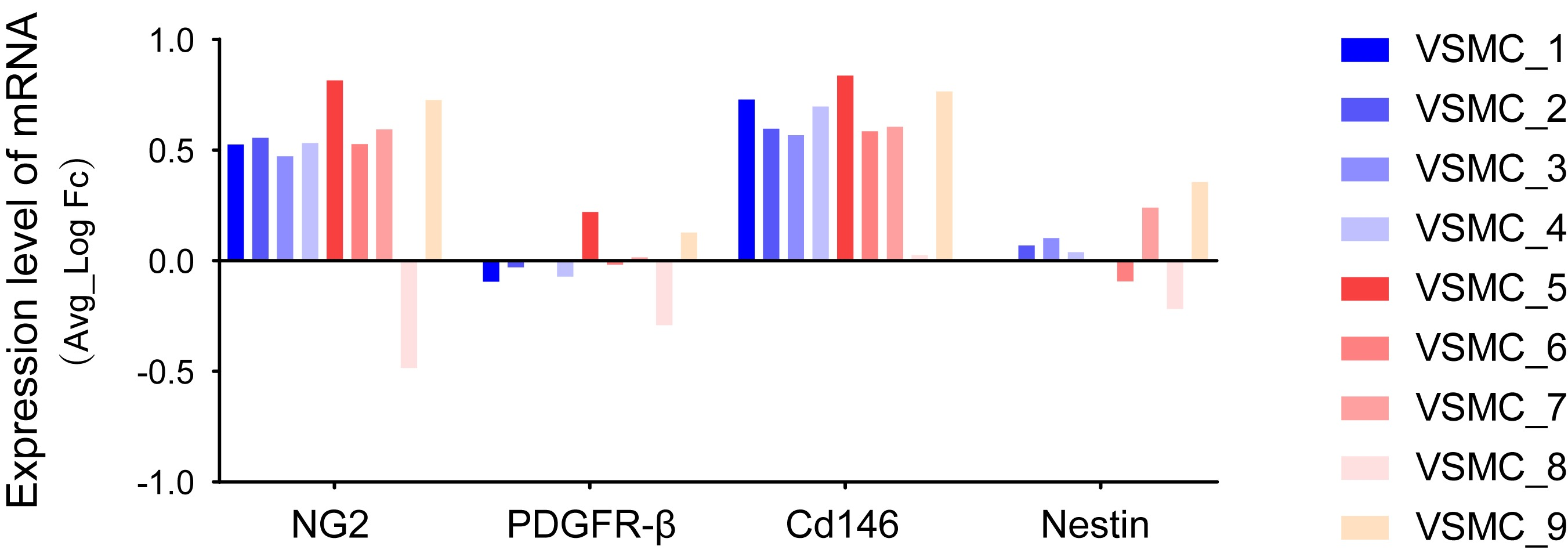

Supplement: S3 Fig — (TIF) [file pone.0265132.s003.tif]

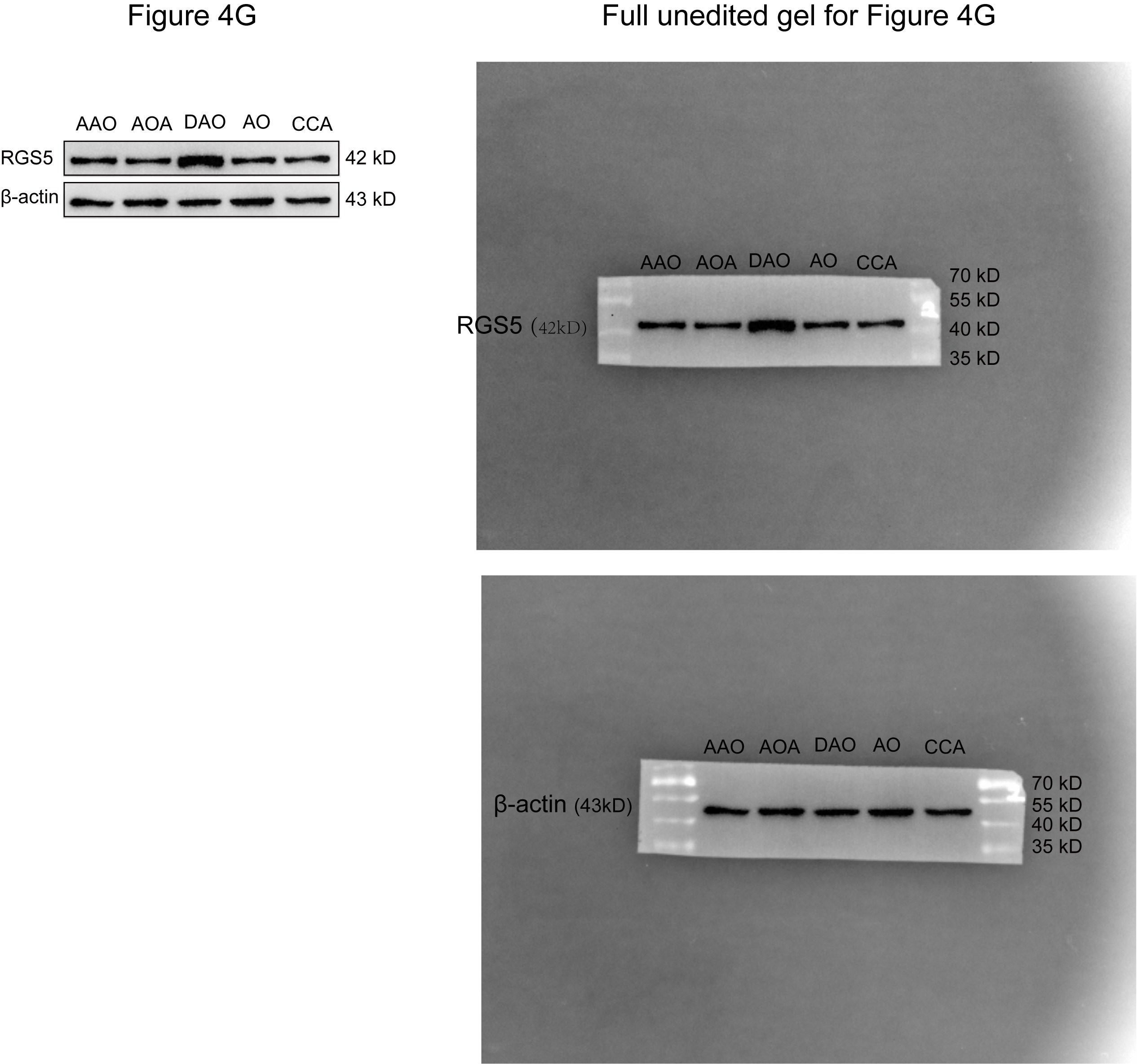

Supplement: S1 Raw data — (TIF) [file pone.0265132.s013.tif]
