# Supplementary material for: A regulator of G protein signaling 5 marked subpopulation of vascular smooth muscle cells is lost during vascular disease
Source: PLoS One. 2022 Mar 23;17(3):e0265132. doi: 10.1371/journal.pone.0265132 (PMC8942229; doi:10.1371/journal.pone.0265132)
Supplement: S1 File — (PDF) [file pone.0265132.s004.pdf]

Differentially expressed genes in VSMC_1 cluster

gene	*p*_val	avg_logFC	pct.1	pct.2
Fibin	3.05E-215	0.495784396	0.978	0.828
Net1	9.89E-205	0.424434837	0.975	0.81
Atf3	8.72E-168	0.40889765	0.843	0.553
Id1	1.86E-126	0.390259266	0.95	0.82
Fos	2.31E-222	0.388578518	1	0.962
Btg2	2.50E-129	0.379695815	0.988	0.893
Slc22a1	2.17E-120	0.336818757	0.654	0.383
Gadd45g	1.22E-64	0.281552846	0.865	0.734
Cnn3	1.08E-118	0.281192629	0.976	0.88
Rock1	4.28E-144	0.274479175	1	0.97
Cyr61	1.78E-116	0.274118287	0.984	0.916
Dnajb4	7.81E-130	0.272450008	0.985	0.896
Ctgf	4.06E-86	0.269178035	0.999	0.966
Nr4a2	1.97E-101	0.260858316	0.704	0.453
Hspa8	1.33E-141	0.258197895	1	0.97

“gene”:the name of each differentially expressed gene.
“*p*_val”: *p* value of significance test. If there are too many decimal places, 0 will be displayed;
“avg_logFC”: fold change of gene average expression level.
“pct.1”: the proportion of cells expressing this gene of particular cluster.
“pct.2”: the proportion of cells expressing this gene of the rest subpopulations.
