# Supplementary material for: A regulator of G protein signaling 5 marked subpopulation of vascular smooth muscle cells is lost during vascular disease
Source: PLoS One. 2022 Mar 23;17(3):e0265132. doi: 10.1371/journal.pone.0265132 (PMC8942229; doi:10.1371/journal.pone.0265132)
Supplement: S2 File — (PDF) [file pone.0265132.s005.pdf]

Differentially expressed genes in VSMC_2 cluster

gene	p_val	avg_logFC	pct.1	pct.2
2210407C18Rik	1.32E-270	0.790440945	0.603	0.205
Cfh	0	0.760299048	0.777	0.347
Nov	1.74E-256	0.621367413	0.985	0.9
Sparcl1	2.74E-143	0.562206528	0.87	0.641
Mfap4	8.54E-113	0.507899851	0.909	0.761
Hand2	0	0.498951274	0.457	0.023
Tnnt2	1.66E-63	0.482511356	0.281	0.127
Aspn	2.24E-119	0.459449964	0.413	0.169
Bmp3	5.58E-114	0.445200172	0.626	0.37
Npy1r	1.01E-160	0.442165929	0.932	0.749
Wif1	2.51E-88	0.422029453	0.636	0.418
Lpl	1.42E-233	0.418870427	0.464	0.13
Svil	7.95E-143	0.384705503	0.919	0.759
Col14a1	1.12E-128	0.375670451	0.558	0.266
Kcnj15	7.74E-166	0.36119544	0.438	0.148
Igfbp4	8.01E-179	0.353198044	0.365	0.093
Sfrp2	2.20E-75	0.333541487	0.639	0.407
Sphkap	2.49E-238	0.332216514	0.358	0.068
Tcap	1.85E-31	0.331689393	0.889	0.81
Cped1	1.49E-140	0.330036011	0.997	0.945
Fbln5	2.92E-107	0.327686014	0.985	0.912
Hspb6	3.81E-91	0.321679913	0.841	0.669
Csrp2	4.59E-96	0.302847602	0.998	0.987
Dkk3	9.06E-87	0.29894071	0.887	0.73
Mylk4	4.16E-81	0.287236343	0.972	0.858
Acan	1.59E-54	0.284377215	0.372	0.204
Fbxl22	2.81E-46	0.281532202	0.875	0.764
Col15a1	1.11E-75	0.280705182	0.826	0.641
Tbx20	9.58E-226	0.280264017	0.349	0.068
Tcf4	5.19E-98	0.279973322	0.709	0.448
Uchl1	2.30E-72	0.273276942	0.784	0.603
Aebp1	2.26E-82	0.271633706	0.996	0.967
Sost	8.52E-43	0.271541991	0.989	0.937
Lmo4	3.05E-71	0.258673004	0.697	0.501
Gpx1	1.84E-82	0.256192928	0.953	0.901
Ace	4.49E-85	0.255648748	0.675	0.436
Gxylt2	1.15E-95	0.253451197	0.669	0.411
Sod3	1.48E-88	0.252595115	0.977	0.91
Ecm1	6.24E-67	0.250064166	0.954	0.847

“gene”:the name of each differentially expressed gene.

"*p*_val": *p* value of significance test. If there are too many decimal places, 0 will be displayed;
"avg_logFC": fold change of gene average expression level.
"pct.1": the proportion of cells expressing this gene of particular cluster.
"pct.2": the proportion of cells expressing this gene of the rest subpopulations.
