# Supplementary material for: A regulator of G protein signaling 5 marked subpopulation of vascular smooth muscle cells is lost during vascular disease
Source: PLoS One. 2022 Mar 23;17(3):e0265132. doi: 10.1371/journal.pone.0265132 (PMC8942229; doi:10.1371/journal.pone.0265132)
Supplement: S3 File — (PDF) [file pone.0265132.s006.pdf]

# Differentially expressed genes in VSMC_3 cluster

| gene | p_val | avg_logFC | pct.1 | pct.2 |
|---|---|---|---|---|
| Wisp2 | 5.38E-130 | 0.447626855 | 0.783 | 0.51 |
| Nupr1 | 3.67E-146 | 0.403448719 | 0.991 | 0.933 |
| Tcap | 1.29E-53 | 0.291768918 | 0.927 | 0.804 |
| Mfap5 | 2.91E-64 | 0.287453789 | 0.922 | 0.798 |
| Fth1 | 4.45E-178 | 0.285192676 | 1 | 0.994 |
| Tuba1a | 4.83E-84 | 0.283731035 | 0.858 | 0.663 |
| Rbp1 | 7.39E-103 | 0.277035339 | 0.996 | 0.929 |
| Rbp4 | 7.81E-52 | 0.271732478 | 0.897 | 0.742 |
| Lmod1 | 2.08E-115 | 0.268123892 | 0.994 | 0.947 |
| Tubb5 | 2.58E-77 | 0.267923016 | 0.91 | 0.786 |

"gene":the name of each differentially expressed gene.
"*p*_val": *p* value of significance test. If there are too many decimal places, 0 will be displayed;
"avg_logFC": fold change of gene average expression level.
"pct.1": the proportion of cells expressing this gene of particular cluster.
"pct.2": the proportion of cells expressing this gene of the rest subpopulations.
