# Supplementary material for: A regulator of G protein signaling 5 marked subpopulation of vascular smooth muscle cells is lost during vascular disease
Source: PLoS One. 2022 Mar 23;17(3):e0265132. doi: 10.1371/journal.pone.0265132 (PMC8942229; doi:10.1371/journal.pone.0265132)
Supplement: S4 File — (PDF) [file pone.0265132.s007.pdf]

Differentially expressed genes in VSMC_4 cluster

gene	p_val	avg_logFC	pct.1	pct.2
Art3	1.51E-28	0.279404461	0.633	0.474
Emb	1.44E-48	0.27827441	0.83	0.653
Sost	8.66E-56	0.277796582	0.994	0.942
Fibin	3.30E-40	0.277197615	0.965	0.85

"gene":the name of each differentially expressed gene.
"*p*_val": *p* value of significance test. If there are too many decimal places, 0 will be displayed;
"avg_logFC": fold change of gene average expression level.
"pct.1": the proportion of cells expressing this gene of particular cluster.
"pct.2": the proportion of cells expressing this gene of the rest subpopulations.
