# Supplementary material for: A regulator of G protein signaling 5 marked subpopulation of vascular smooth muscle cells is lost during vascular disease
Source: PLoS One. 2022 Mar 23;17(3):e0265132. doi: 10.1371/journal.pone.0265132 (PMC8942229; doi:10.1371/journal.pone.0265132)
Supplement: S5 File — (PDF) [file pone.0265132.s008.pdf]

Differentially expressed genes in VSMC_5 cluster

gene	p_val	avg_logFC	pct.1	pct.2
Hspa1b	1.37E-80	1.250421518	0.862	0.796
Hspa1a	2.56E-50	0.958627224	0.863	0.849
Nr4a1	2.53E-94	0.897750028	0.891	0.795
mt-Co3	7.10E-213	0.888754079	1	0.998
mt-Nd2	2.66E-212	0.872455665	1	0.992
mt-Atp6	1.19E-229	0.863750633	1	0.998
Fosb	2.36E-79	0.862329998	0.907	0.822
Egr1	8.83E-100	0.844851713	0.909	0.829
mt-Co2	7.61E-234	0.831406754	1	0.996
mt-Cytb	1.91E-239	0.82038891	1	0.995
mt-Nd4	4.56E-233	0.80749367	1	0.996
Atf3	1.66E-43	0.805770841	0.735	0.61
mt-Nd5	1.73E-200	0.75929577	1	0.982
mt-Co1	5.67E-247	0.755904486	1	0.997
Klf4	2.44E-38	0.728982463	0.724	0.617
mt-Nd1	1.06E-231	0.717978282	1	0.991
mt-Nd4l	2.29E-211	0.714004246	0.999	0.978
mt-Nd3	1.33E-177	0.702886141	0.995	0.975
Cyr61	3.78E-85	0.702544189	0.957	0.93
Fos	8.19E-87	0.662766173	0.989	0.969
Slc38a2	1.54E-66	0.624312202	0.942	0.93
Jun	1.94E-48	0.588737205	0.93	0.927
Hspg2	7.79E-101	0.580698127	0.954	0.929
Adamts1	1.14E-32	0.57511664	0.845	0.825
Lamb2	4.14E-102	0.573377158	0.966	0.957
Dusp1	1.27E-65	0.557020967	0.954	0.917
Ltbp4	1.37E-90	0.553455745	0.976	0.961
Nr4a2	2.80E-21	0.544785557	0.601	0.504
Cspg4	5.58E-71	0.540101042	0.915	0.908
Pkd1	2.32E-77	0.535624835	0.91	0.883
Irs2	1.63E-23	0.530555868	0.555	0.462
Ddx5	1.32E-98	0.528453406	0.992	0.99
Dnajb1	6.88E-10	0.525204746	0.466	0.415
Zfp36	1.02E-34	0.515479536	0.794	0.752
Col18a1	1.12E-95	0.51158937	0.993	0.971
mt-Atp8	3.39E-53	0.504466842	0.831	0.8
Col1a1	2.76E-64	0.501390215	0.914	0.892
Ier5	2.70E-15	0.488115538	0.517	0.443
Neat1	1.03E-37	0.477567924	0.831	0.794
Ctgf	2.23E-45	0.476640543	0.985	0.972
Col1a2	1.22E-93	0.476071214	0.983	0.968

Fus	1.60E-54	0.470398037	0.884	0.891
Scube3	1.03E-30	0.46889941	0.727	0.703
Malat1	2.44E-138	0.46834081	1	0.999
Tnxb	2.78E-27	0.46795332	0.672	0.601
Junb	8.84E-28	0.467824071	0.801	0.758
AY036118	2.76E-34	0.465973942	0.624	0.492
Lrp1	5.15E-53	0.462224277	0.867	0.856
Fat1	1.10E-47	0.461986362	0.82	0.798
Col3a1	2.57E-63	0.454095503	0.993	0.963
AC149090.1	7.72E-22	0.444327027	0.664	0.635
Col4a2	1.51E-49	0.44387585	0.882	0.91
Dnajb4	4.10E-44	0.443682827	0.93	0.915
Clk1	3.54E-43	0.442138792	0.79	0.756
Ltbp3	1.04E-49	0.433386778	0.855	0.854
Ccnl1	1.00E-23	0.431978489	0.663	0.608
Adcy5	3.40E-53	0.430630048	0.95	0.934
Pcdh7	1.07E-86	0.428096481	0.988	0.968
Ntrk3	2.16E-24	0.426697655	0.644	0.571
Dmd	1.34E-54	0.426567952	0.953	0.952
Sgk1	3.49E-13	0.425548004	0.637	0.618
Socs3	5.20E-07	0.421792977	0.312	0.25
Lnpep	2.24E-35	0.418552657	0.832	0.839
Myom1	4.62E-47	0.41084062	0.902	0.88
Nfkbiz	2.72E-17	0.409830386	0.499	0.416
Ubc	4.56E-30	0.409203319	0.976	0.984
Eln	2.94E-36	0.40833568	0.94	0.936
Itga9	5.24E-45	0.40557982	0.878	0.884
Hnrnph1	1.39E-44	0.404714715	0.868	0.871
Col6a3	1.86E-29	0.402777975	0.758	0.743
Klf2	7.92E-17	0.402060126	0.704	0.69
Trio	1.19E-24	0.400418649	0.668	0.618
Klf6	1.36E-27	0.399489725	0.851	0.892
Gm42418	4.65E-64	0.398540413	1	0.998
Per2	2.04E-18	0.398493498	0.554	0.483
Son	1.05E-48	0.398222813	0.94	0.96
Thsd4	1.11E-33	0.3941164	0.828	0.815
Wwp2	1.29E-45	0.393545314	0.89	0.895
Mcam	5.96E-37	0.389095915	0.925	0.909
Sacs	2.40E-14	0.386513314	0.519	0.458
Fbn1	3.68E-24	0.384543169	0.708	0.7
Gadd45g	1.04E-11	0.382080872	0.728	0.768
Adgrl1	4.69E-17	0.381818322	0.617	0.585
Arrdc3	8.38E-09	0.378916319	0.556	0.535
Sorl1	1.87E-25	0.378573512	0.788	0.808

Myo9b	8.20E-17	0.37699828	0.539	0.468
Otud1	1.09E-10	0.374874409	0.687	0.704
Gm26917	5.08E-22	0.373686507	0.616	0.533
Itga5	6.60E-09	0.373675915	0.565	0.541
Lama5	2.60E-14	0.373184201	0.505	0.427
Btg1	5.51E-16	0.372158977	0.758	0.812
Npr1	5.81E-22	0.371687941	0.743	0.742
Atp2a2	1.60E-49	0.367740942	0.964	0.954
Col4a1	2.29E-44	0.367207849	0.922	0.946
mt-Nd6	4.21E-19	0.36524234	0.397	0.276
Col5a1	4.98E-24	0.365218754	0.68	0.648
Itga7	6.82E-14	0.365064072	0.601	0.561
Kcnq1ot1	2.32E-10	0.365024974	0.453	0.383
Smad7	1.12E-19	0.363767126	0.754	0.781
Unc5c	3.59E-16	0.363386106	0.548	0.471
Tgfbr1	6.88E-12	0.361159074	0.601	0.586
Ccnl2	1.09E-11	0.359453541	0.595	0.578
Tsc22d2	1.47E-08	0.359370847	0.567	0.562
Nav2	1.56E-12	0.357828105	0.583	0.56
Col4a5	9.90E-32	0.353855281	0.809	0.822
Atp2b4	5.18E-25	0.350627279	0.801	0.822
Nktr	1.31E-18	0.34965204	0.716	0.748
Lama4	1.28E-15	0.34921074	0.679	0.676
Chka	8.86E-07	0.347532461	0.435	0.402
Agrn	2.09E-14	0.341506096	0.429	0.34
Itga8	2.85E-64	0.340994279	0.995	0.972
Auts2	4.61E-25	0.340604676	0.774	0.796
Ptprs	1.09E-24	0.340466408	0.714	0.699
Luc7l2	1.00E-25	0.338490887	0.837	0.895
Ptgs2	0.003719184	0.337664643	0.254	0.221
Serpine1	2.27E-12	0.336742762	0.72	0.734
Enah	2.59E-28	0.335051856	0.929	0.933
Tra2b	3.79E-11	0.333206852	0.716	0.772
Sun2	1.21E-24	0.330298027	0.813	0.839
Igf1r	3.92E-16	0.329684174	0.691	0.7
Nisch	1.11E-17	0.327767674	0.751	0.783
Csrnp1	6.04E-12	0.326781912	0.327	0.239
Clstn1	2.38E-14	0.325827182	0.681	0.693
Ppp1r15a	4.26E-10	0.325416148	0.652	0.656
Hsph1	0.006030424	0.32451915	0.569	0.654
Igsf9b	8.72E-09	0.324150672	0.431	0.387
Myh9	3.53E-19	0.323871385	0.698	0.705
Tra2a	6.04E-15	0.322927864	0.707	0.744
Zbtb20	7.11E-43	0.321428391	0.98	0.977

Snrnp70	2.54E-20	0.32108971	0.784	0.825
Itgb5	5.99E-32	0.320972164	0.87	0.907
Pde4dip	5.48E-16	0.318572042	0.738	0.795
Plin4	3.09E-17	0.317476449	0.738	0.774
Col6a1	2.15E-25	0.317190896	0.773	0.795
2810474O19Rik	0.000144559	0.317142583	0.62	0.673
Errfi1	0.000273958	0.315771821	0.547	0.564
Sf3b1	1.29E-28	0.315135172	0.841	0.876
Chd6	1.29E-16	0.314482201	0.773	0.814
Ptprd	1.66E-09	0.314322808	0.633	0.651
Lgr6	3.41E-15	0.313205054	0.688	0.693
Pdgfrb	1.74E-13	0.311693867	0.61	0.591
Nfat5	1.15E-14	0.311223696	0.652	0.652
Per1	5.84E-08	0.307032722	0.472	0.435
Postn	8.14E-33	0.305111943	0.96	0.94
Notch2	1.04E-07	0.303948947	0.493	0.473
Dst	8.00E-41	0.300528041	0.961	0.972
Ano1	3.18E-11	0.300448764	0.46	0.389
Trp53inp1	1.84E-05	0.300015392	0.556	0.582
Grip2	8.53E-09	0.299808104	0.363	0.299
Ints6l	6.05E-08	0.299627005	0.351	0.29
Smg1	4.20E-08	0.298861099	0.497	0.475
Itga3	0.000370915	0.297820787	0.434	0.428
Ank2	6.03E-09	0.297387974	0.577	0.569
Fryl	5.26E-14	0.295712884	0.641	0.634
Thrb	1.20E-05	0.295380613	0.53	0.545
Prpf39	1.33E-07	0.294699419	0.453	0.406
Camk2d	1.58E-12	0.294552183	0.727	0.804
Ryr2	1.62E-07	0.294493543	0.477	0.453
Cebpb	1.09E-08	0.293385321	0.737	0.807
Smoc1	3.52E-15	0.293253679	0.718	0.744
Skil	3.85E-08	0.292877695	0.63	0.692
Adam33	1.24E-08	0.292395943	0.591	0.591
Srsf11	4.08E-16	0.290432329	0.741	0.787
Glg1	1.51E-20	0.289112382	0.794	0.839
Abcc1	2.78E-08	0.287507209	0.528	0.509
Col4a6	3.36E-12	0.287231174	0.616	0.611
Hk2	2.57E-07	0.287112483	0.45	0.399
Nbeal1	2.98E-20	0.286691711	0.823	0.879
Meg3	0.000285515	0.286179035	0.483	0.465
Ltbp2	4.55E-06	0.286165791	0.352	0.305
Gadd45b	0.010164148	0.285988169	0.626	0.71
Rasl11b	0.000994246	0.285071995	0.57	0.609
Col15a1	9.17E-14	0.28487731	0.667	0.677

Col5a2	5.93E-20	0.284796321	0.786	0.815
Ier2	4.64E-14	0.282820916	0.72	0.701
Bcam	6.82E-22	0.282771311	0.913	0.941
Tob1	2.74E-05	0.28275402	0.495	0.484
Nufip2	3.99E-05	0.282544322	0.484	0.475
Mical3	1.25E-07	0.281419697	0.5	0.475
Ptp4a1	5.27E-05	0.280782076	0.558	0.596
Fbxo30	3.91E-09	0.280675301	0.659	0.68
Ntn1	3.31E-06	0.279234483	0.62	0.67
Pde3a	8.08E-20	0.279223725	0.789	0.823
Srsf2	6.87E-16	0.278577712	0.777	0.837
Pnisr	4.28E-10	0.277023225	0.758	0.83
Trpm7	7.83E-09	0.276436911	0.626	0.652
Ppp1r12b	3.38E-24	0.276068034	0.898	0.925
Srsf5	2.93E-20	0.276014659	0.847	0.891
Wsb1	7.94E-05	0.274605898	0.378	0.348
Plekha1	1.25E-06	0.274093295	0.547	0.539
Phip	4.55E-08	0.273408482	0.605	0.615
Rbm5	5.85E-08	0.272059371	0.61	0.62
Ltbp1	5.29E-25	0.270697024	0.906	0.932
Fosl2	0.001362482	0.269727618	0.448	0.456
Flna	2.81E-58	0.269211065	1	0.992
Tmtc1	1.32E-06	0.26815572	0.601	0.626
Mast4	6.39E-06	0.267977411	0.602	0.628
Lamc1	0.000362999	0.267869515	0.551	0.582
Ranbp2	6.47E-09	0.267312252	0.637	0.669
Plekhg3	1.69E-08	0.26730743	0.622	0.647
Aoc3	2.28E-12	0.266682025	0.784	0.84
Herc1	2.31E-05	0.266367128	0.513	0.507
Lrp10	1.80E-05	0.266000102	0.527	0.529
Adamts10	8.82E-08	0.26530296	0.496	0.464
Prrc2b	2.37E-08	0.264882435	0.651	0.68
Plxnb2	1.56E-05	0.264791415	0.474	0.465
Pkdcc	1.75E-07	0.26422935	0.673	0.702
Axl	1.14E-10	0.263734846	0.755	0.833
Irf2bp2	4.40E-07	0.262905146	0.668	0.724
Hip1	3.75E-07	0.262839197	0.727	0.797
Krit1	0.001609374	0.261865124	0.543	0.581
Abcc9	1.14E-06	0.261545355	0.659	0.711
Xpr1	2.87E-07	0.261318	0.637	0.687
Slc1a5	1.41E-05	0.260419678	0.559	0.58
Hbegf	0.240520152	0.260332145	0.47	0.505
Srrm2	7.63E-24	0.25978218	0.883	0.932
4932438A13Rik	3.44E-08	0.259478275	0.495	0.468

Fam126b	0.001985541	0.258850809	0.539	0.573
Btg2	6.46E-16	0.258681101	0.907	0.916
Slc4a3	0.001980795	0.257414245	0.457	0.472
Ncam1	1.74E-26	0.25703611	0.922	0.943
Tacc2	1.68E-11	0.256151829	0.691	0.739
Atp1a2	1.92E-13	0.255566233	0.836	0.868
Taf1d	0.000130887	0.255082265	0.411	0.384
Lyst	1.18E-05	0.255013642	0.474	0.455
Esyt2	1.91E-22	0.254278612	0.82	0.856
Nrbp2	0.014063864	0.254085247	0.351	0.346
Ubp1	0.000720373	0.253659015	0.481	0.486
Ifrd1	0.001567091	0.252376833	0.585	0.644
Creb5	3.07E-05	0.252373218	0.59	0.612
Zcchc14	0.000272004	0.251257927	0.435	0.43
Amotl2	0.036653137	0.251144713	0.414	0.431
Kank1	1.39E-14	0.250461205	0.805	0.875
Vasn	6.90E-11	0.250136612	0.762	0.829
Gm26632	1.32E-06	0.250099456	0.358	0.307

"gene":the name of each differentially expressed gene.
"*p*_val": *p* value of significance test. If there are too many decimal places, 0 will be displayed;
"avg_logFC": fold change of gene average expression level.
"pct.1": the proportion of cells expressing this gene of particular cluster.
"pct.2": the proportion of cells expressing this gene of the rest subpopulations.
