# Supplementary material for: A regulator of G protein signaling 5 marked subpopulation of vascular smooth muscle cells is lost during vascular disease
Source: PLoS One. 2022 Mar 23;17(3):e0265132. doi: 10.1371/journal.pone.0265132 (PMC8942229; doi:10.1371/journal.pone.0265132)
Supplement: S6 File — (PDF) [file pone.0265132.s009.pdf]

Differentially expressed genes in VSMC_6 cluster

gene	p_val	avg_logFC	pct.1	pct.2
Rgs5	0	2.107691752	0.983	0.713
Fn1	5.97E-274	1.488535691	0.836	0.284
Igfbp5	1.57E-286	1.454468448	0.998	0.945
Des	6.52E-118	1.100056583	0.569	0.212
Palld	3.15E-193	1.034354255	0.909	0.547
Timp4	4.10E-184	0.746626699	0.605	0.174
Ndrg1	1.26E-138	0.701000668	0.806	0.423
Hmgcs2	6.93E-88	0.632060702	0.731	0.441
Kit	0	0.594723721	0.454	0.036
Synm	1.40E-119	0.584926123	0.808	0.481
P2rx1	2.84E-135	0.580182305	0.548	0.171
Vegfd	1.69E-165	0.558913178	0.592	0.176
Mustn1	7.86E-42	0.531794871	0.893	0.776
Hoxa10	0	0.521372863	0.498	0.01
Tmem176b	1.92E-208	0.503531144	0.493	0.089
Tes	5.56E-81	0.502589062	0.797	0.534
Pkdcc	1.29E-69	0.495817315	0.876	0.686
Txnip	7.47E-66	0.486302773	0.932	0.827
Fbln7	0	0.481431111	0.43	0.035
Hoxc10	0	0.445778638	0.4	0.004
Hoxa9	0	0.441659948	0.391	0.019
Map3k7cl	7.58E-61	0.43827848	0.817	0.621
Parm1	3.91E-73	0.402553971	0.57	0.28
Crip1	3.16E-50	0.400488003	0.978	0.943
Rnd3	1.39E-33	0.390407511	0.688	0.509
Pi15	1.48E-52	0.385238909	0.876	0.75
Btc	3.56E-59	0.380317241	0.521	0.262
Rgs2	4.41E-25	0.373527018	0.51	0.34
Ccdc68	1.43E-71	0.373313326	0.609	0.318
Adamtsl3	2.06E-62	0.369147422	0.535	0.266
Col4a2	1.46E-54	0.360104416	0.957	0.904
Nkx3-1	4.03E-210	0.357240789	0.224	0.013
Tnc	1.88E-56	0.356982028	0.565	0.303
Adgrl2	3.19E-39	0.341530912	0.806	0.662
Sparcl1	6.38E-09	0.335683292	0.72	0.681
Cdh11	3.91E-153	0.323984188	0.318	0.049
Bche	1.69E-71	0.32382558	0.417	0.154
Serpine2	1.05E-56	0.320761723	0.997	0.96
Slc6a6	1.17E-39	0.318533513	0.803	0.638
Sorbs2	8.26E-07	0.315859403	0.619	0.614
Gm13861	8.71E-45	0.310852492	0.454	0.228

Adamts15	2.69E-49	0.305663443	0.389	0.167
Pax1	7.95E-209	0.305470921	0.236	0.016
Col19a1	4.58E-109	0.304784847	0.373	0.093
Crispld2	1.07E-26	0.301155713	0.783	0.65
Nampt	6.88E-39	0.300803089	0.687	0.478
Nrtn	7.15E-36	0.290433274	0.646	0.432
Slc7a2	1.61E-58	0.290160926	0.449	0.2
Nr2f2	1.86E-29	0.288938427	0.677	0.528
Btg2	3.05E-14	0.28847792	0.95	0.913
Mtss1l	1.91E-46	0.28332692	0.531	0.304
Hoxc8	3.17E-301	0.27635349	0.254	0.009
Hoxc6	3.43E-138	0.27586603	0.282	0.042
Adamts8	5.02E-25	0.275172766	0.411	0.253
Cdc42ep3	6.87E-31	0.275031249	0.896	0.811
Meis1	3.91E-37	0.274539826	0.488	0.286
Epha3	1.03E-34	0.266638236	0.428	0.235
Cryab	2.78E-21	0.265564916	0.956	0.89
Ptger3	1.18E-167	0.260273578	0.272	0.031
Hoxc9	0	0.259451973	0.252	0.007
Nov	2.46E-23	0.258845871	0.97	0.912
Rbp4	0.00698609	0.258604094	0.72	0.773
Gm13889	2.01E-17	0.256906137	0.85	0.775
Pik3r1	4.60E-25	0.255927262	0.69	0.543
Fabp4	3.52E-16	0.253987301	0.82	0.728

"gene":the name of each differentially expressed gene.
"*p*_val": *p* value of significance test. If there are too many decimal places, 0 will be displayed;
"avg_logFC": fold change of gene average expression level.
"pct.1": the proportion of cells expressing this gene of particular cluster.
"pct.2": the proportion of cells expressing this gene of the rest subpopulations.
