# Supplementary material for: A regulator of G protein signaling 5 marked subpopulation of vascular smooth muscle cells is lost during vascular disease
Source: PLoS One. 2022 Mar 23;17(3):e0265132. doi: 10.1371/journal.pone.0265132 (PMC8942229; doi:10.1371/journal.pone.0265132)
Supplement: S7 File — (PDF) [file pone.0265132.s010.pdf]

Differentially expressed genes in VSMC_7 cluster

gene	p_val	avg_logFC	pct.1	pct.2
Prss23	1.10E-138	0.840379909	0.94	0.656
S100a4	8.13E-95	0.830271537	0.903	0.636
Itga5	3.14E-129	0.74983905	0.905	0.519
S100a10	1.14E-106	0.730980255	0.861	0.556
Anxa2	7.46E-132	0.695065319	0.956	0.734
S100a6	1.16E-157	0.693276005	0.996	0.96
Eln	2.64E-61	0.693223402	0.991	0.932
Vcam1	4.01E-32	0.687714034	0.232	0.083
Mustn1	4.64E-69	0.668038124	0.951	0.773
Mgp	4.43E-22	0.649028156	1	1
Fn1	2.35E-96	0.645938619	0.993	0.946
Anxa1	2.54E-114	0.643760468	0.965	0.779
Tnfrsf12a	1.74E-88	0.62516714	0.936	0.666
Lgals3	2.03E-165	0.621075999	0.549	0.131
Timp1	2.19E-43	0.575853185	0.259	0.085
Tspo	8.45E-84	0.48839549	0.978	0.866
Cd109	8.34E-99	0.451494919	0.575	0.207
Flnc	1.18E-56	0.450372612	0.776	0.508
Emp3	9.69E-88	0.449601608	0.976	0.874
Lgals1	4.78E-100	0.447194084	0.996	0.968
Postn	7.50E-60	0.433641004	0.995	0.938
Fxyd5	7.40E-87	0.432930763	0.578	0.229
Col1a1	3.23E-44	0.413036196	0.969	0.889
Tagln2	1.26E-50	0.397153955	0.985	0.907
Cyb5r3	1.27E-58	0.389140011	0.973	0.894
Msn	1.25E-58	0.385041263	0.985	0.906
Tuba1a	1.16E-37	0.375906122	0.881	0.685
Rtn4	3.78E-49	0.366791047	0.947	0.876
Rexo2	5.82E-64	0.365011334	0.98	0.87
Ppp1r14b	2.20E-47	0.356322543	0.659	0.378
Cd81	3.03E-65	0.341447764	0.991	0.951
Tmsb10	3.98E-38	0.339380059	0.825	0.626
Col15a1	1.62E-43	0.338549038	0.865	0.663
Ahnak2	8.69E-39	0.334420514	0.759	0.533
Hspb7	1.20E-48	0.329103877	0.96	0.822
Fhl1	7.59E-36	0.32515451	0.989	0.967
S100a11	2.10E-60	0.323389886	0.989	0.932
Crip1	2.94E-34	0.32317141	0.993	0.942
Uchl1	9.80E-36	0.322992907	0.836	0.624
Nupr1	5.22E-14	0.321794675	0.982	0.941
Gm13889	1.08E-22	0.294464233	0.901	0.773
Lmna	1.11E-37	0.292649888	0.987	0.942
Tgm2	2.95E-33	0.281671108	0.945	0.836
Prnp	3.96E-33	0.277734501	0.741	0.516
Nes	2.04E-21	0.274750238	0.75	0.58

Tm4sf1	1.61E-26	0.274612118	0.993	0.954
Anxa5	2.49E-36	0.274194518	0.96	0.868
Cd63	1.51E-46	0.273912435	0.993	0.932
Tubb2a	1.90E-41	0.270914134	0.538	0.282
Cdo1	2.27E-20	0.269698127	0.823	0.643
Fabp4	1.88E-16	0.261532872	0.856	0.727
Pfn1	4.91E-40	0.254525087	0.991	0.939
Ehd2	1.82E-36	0.253460406	0.918	0.778
Tgfb2	1.95E-24	0.251754011	0.774	0.598
Dap	4.74E-32	0.250772028	0.854	0.662
Tubb4b	4.14E-17	0.2502211	0.803	0.642

“gene”:the name of each differentially expressed gene.
“p_val”: p value of significance test. If there are too many decimal places, 0 will be displayed;
“avg_logFC”: fold change of gene average expression level.
“pct.1”: the proportion of cells expressing this gene of particular cluster.
“pct.2”: the proportion of cells expressing this gene of the rest subpopulations.
