# Supplementary material for: A regulator of G protein signaling 5 marked subpopulation of vascular smooth muscle cells is lost during vascular disease
Source: PLoS One. 2022 Mar 23;17(3):e0265132. doi: 10.1371/journal.pone.0265132 (PMC8942229; doi:10.1371/journal.pone.0265132)
Supplement: S8 File — (PDF) [file pone.0265132.s011.pdf]

Differentially expressed genes in VSMC_8 cluster

gene	p_val	avg_logFC	pct.1	pct.2
Apoe	2.13E-06	1.591641589	0.155	0.348
Gm42418	1.34E-98	1.374258752	0.985	0.998
AY036118	9.05E-08	1.195009596	0.481	0.504
Gm26917	0.380655998	1.173156397	0.348	0.546
Hspa1b	0.000320949	1.082401726	0.432	0.813
Nr4a1	0.930412644	1.019895495	0.496	0.813
Hspa1a	0.002140175	0.986861227	0.47	0.861
Atf3	0.20966436	0.863541802	0.394	0.627
Fosb	0.08348676	0.843107185	0.572	0.837
Neat1	0.013119369	0.833857523	0.428	0.808
Ftl1	1.14E-13	0.815678473	0.348	0.886
Myom1	0.007148924	0.78627641	0.439	0.895
Zfp36	0.000395252	0.774087334	0.386	0.767
Ptgs2	6.01E-05	0.767958698	0.102	0.228
AC149090.1	1.30E-07	0.76450378	0.292	0.648
Ddx5	5.72E-19	0.755463832	0.799	0.996
Egr1	0.705685747	0.724033812	0.553	0.844
Cyr61	0.064775069	0.683407597	0.652	0.94
Mafb	0.114469868	0.681984458	0.068	0.106
Pim1	0.016856561	0.680812397	0.121	0.2
Sfswap	2.20E-08	0.676653008	0.205	0.476
Junb	0.000525634	0.669160454	0.402	0.773
Dusp1	0.024413579	0.663692699	0.606	0.93
Son	0.037191036	0.638500964	0.598	0.97
Nfkbiz	2.63E-07	0.623298357	0.193	0.43
Lars2	0.0206702	0.623203922	0.466	0.899
Adamts10	1.33E-08	0.619774834	0.197	0.475
Nr4a2	3.08E-10	0.618322449	0.216	0.521
Snrnp70	2.96E-08	0.605569882	0.375	0.835
Jun	0.475331444	0.591143585	0.625	0.936
Ppp1r15a	1.93E-14	0.586233879	0.258	0.668
Pnisr	1.68E-05	0.569321236	0.409	0.837
Ccnl1	1.08E-08	0.565322804	0.273	0.623
Srsf5	0.000407283	0.561621637	0.443	0.901
Nfat5	7.70E-09	0.559982878	0.295	0.663
Klf4	2.78E-10	0.551897616	0.28	0.637
Ier2	0.007744471	0.550832825	0.402	0.712
Hes1	1.73E-08	0.548745926	0.174	0.413
Ntrk3	5.16E-12	0.547110731	0.227	0.588
Rgs2	9.75E-07	0.54609857	0.159	0.358
Clk1	6.94E-09	0.543301727	0.333	0.772

Ubc	2.59E-08	0.539803317	0.746	0.991
Wwp2	9.06E-07	0.538157378	0.405	0.909
Myh9	1.11E-08	0.533772474	0.318	0.716
Pkd1	1.70E-05	0.526964233	0.417	0.9
Cdkn1a	0.167858653	0.526390342	0.087	0.124
Myo9b	4.74E-11	0.525078554	0.189	0.483
Nisch	8.85E-12	0.52494341	0.33	0.795
Prpf4b	5.03E-11	0.52187256	0.311	0.745
Ier5	9.02E-09	0.517462559	0.189	0.457
Sf3b1	8.41E-06	0.517412106	0.417	0.887
Csrnp1	0.008567389	0.5160395	0.148	0.25
Speg	4.50E-18	0.513740266	0.235	0.691
Srsf11	1.95E-12	0.512087943	0.322	0.797
Nktr	6.77E-11	0.506938079	0.314	0.759
Nfkbia	1.59E-28	0.506632153	0.167	0.649
Ints6l	4.24E-06	0.503498494	0.133	0.3
Ccrl2	1.93E-05	0.501370354	0.057	0.164
Prpf39	3.19E-09	0.496380733	0.17	0.417
Grip2	2.45E-06	0.491374291	0.133	0.31
Ccnl2	4.46E-14	0.490428837	0.216	0.591
Akap13	1.17E-11	0.48700682	0.337	0.816
Stx5a	2.03E-11	0.48447012	0.144	0.415
Dnmt3a	8.26E-12	0.483236653	0.17	0.46
Chd8	3.60E-09	0.47975154	0.163	0.399
Adgrl1	9.06E-14	0.47613678	0.223	0.599
Fus	6.95E-08	0.475890933	0.405	0.905
Mical3	2.14E-14	0.474370692	0.163	0.486
Fos	0.005310618	0.472431462	0.848	0.974
Hnrnph1	1.56E-07	0.471248541	0.402	0.885
Kank1	1.58E-06	0.463247657	0.402	0.883
Adamts1	6.53E-07	0.462045649	0.402	0.839
Arrdc3	2.14E-11	0.459763952	0.22	0.546
Tnrc6a	1.12E-16	0.458529118	0.246	0.677
Luc7l2	4.38E-09	0.454958112	0.398	0.905
Dmd	0.708322392	0.453245356	0.58	0.963
Gm26632	8.86E-07	0.45117165	0.136	0.316
Cabin1	8.47E-10	0.451079425	0.144	0.38
Zcchc14	5.45E-15	0.447353045	0.133	0.44
Kdm6b	2.10E-06	0.446756989	0.136	0.311
Rsrp1	4.41E-08	0.445488901	0.405	0.888
Per2	7.48E-14	0.44455874	0.17	0.498
Sh3bgrl3	1.01E-29	0.444196111	0.174	0.676
Rbm5	1.01E-12	0.443891551	0.246	0.631
Ewsr1	3.52E-16	0.4419138	0.25	0.678

Sacs	3.90E-14	0.441514288	0.155	0.472
Meg3	4.92E-08	0.441358216	0.216	0.474
Zcchc7	5.35E-15	0.440964346	0.208	0.574
Plekhg3	5.12E-16	0.436610784	0.239	0.658
Nfkb1	8.44E-09	0.435501195	0.14	0.351
Prrc2c	2.32E-09	0.432203067	0.375	0.85
Dnajb1	7.45E-10	0.43053842	0.167	0.427
Srrm1	4.58E-08	0.42890053	0.364	0.806
Cspp1	4.93E-09	0.42871237	0.121	0.327
Mical2	4.03E-14	0.426615195	0.284	0.744
Zscan26	1.22E-07	0.423926369	0.129	0.323
Sf1	1.07E-15	0.422108394	0.167	0.508
Fam135a	3.51E-12	0.421674955	0.136	0.413
2410089E03Rik	0.000302406	0.421304943	0.117	0.237
Fxyd5	3.75E-06	0.414726206	0.106	0.255
Phip	8.75E-18	0.414646958	0.212	0.627
Phldb1	1.75E-06	0.407850442	0.121	0.286
Phf1	5.88E-06	0.406713325	0.102	0.247
Cys1	7.57E-18	0.406250344	0.155	0.52
Nav2	3.61E-17	0.405365452	0.193	0.573
Braf	4.32E-15	0.403431831	0.129	0.427
Ccdc88c	0.000268315	0.402461578	0.068	0.16
Srrm2	2.69E-05	0.400598634	0.458	0.942
Maf	1.14E-10	0.399572105	0.152	0.409
Fchsd2	4.23E-18	0.399238052	0.155	0.513
Arhgef2	2.07E-19	0.397622466	0.239	0.696
Large1	2.54E-14	0.395846166	0.148	0.458
Dicer1	2.36E-15	0.394390634	0.14	0.457
Enah	0.291069414	0.393213655	0.542	0.945
Brd4	2.04E-20	0.392221211	0.163	0.557
Slc1a5	7.57E-17	0.39171127	0.201	0.589
Per3	7.27E-15	0.390833858	0.17	0.5
Arhgef25	1.41E-20	0.39061398	0.193	0.629
Dmpk	9.96E-07	0.390332077	0.413	0.907
Dopey1	4.64E-08	0.389335527	0.106	0.282
Clk4	2.89E-11	0.389324354	0.133	0.39
Abi1	2.84E-18	0.380584869	0.159	0.513
Rbm25	2.97E-16	0.379896015	0.314	0.822
Rpgrip1	0.013842479	0.379669925	0.072	0.132
Dtx3	1.85E-17	0.378859237	0.159	0.515
Gadd45b	6.71E-19	0.378728958	0.258	0.717
Ccar1	2.45E-16	0.378242136	0.205	0.585
Ccdc141	1.80E-15	0.378194004	0.133	0.444
Adam33	2.80E-22	0.377953408	0.178	0.604

Lima1	2.16E-05	0.37771419	0.106	0.242
Pnck	3.43E-11	0.376047913	0.102	0.327
Taf1d	7.37E-10	0.373901754	0.155	0.394
Ankrd17	2.43E-14	0.373699309	0.17	0.504
Pid1	1.51E-07	0.372953175	0.121	0.305
Notch2	3.87E-14	0.372673617	0.167	0.484
Ddx17	1.58E-18	0.370962361	0.223	0.647
Hdac7	2.61E-08	0.370855573	0.114	0.306
Abcc5	1.18E-09	0.369687812	0.125	0.343
Acin1	1.67E-16	0.369651118	0.25	0.692
Cebpb	9.14E-16	0.367223567	0.318	0.816
Golgb1	2.01E-14	0.367144611	0.333	0.837
Setd5	1.47E-17	0.366376769	0.148	0.5
Fnbp1	7.27E-09	0.36625841	0.133	0.351
Taok2	6.92E-07	0.364936922	0.091	0.245
7-Mar	9.91E-11	0.364489202	0.174	0.447
Birc6	4.97E-18	0.364226629	0.235	0.673
Huwe1	1.06E-22	0.36384894	0.22	0.69
Epb41	5.92E-15	0.363556565	0.14	0.443
Arhgef1	8.56E-11	0.363493202	0.129	0.369
Slc38a2	0.000739114	0.363005087	0.5	0.945
Bmp1	9.33E-11	0.362733814	0.106	0.333
Malat1	0.844890579	0.362068068	0.985	1
Tnrc6c	6.16E-19	0.361134622	0.265	0.743
Mast4	2.54E-20	0.36078151	0.201	0.639
Atrx	3.58E-14	0.360607828	0.341	0.845
Naa16	0.000756038	0.359212084	0.076	0.162
Ube2d3	6.18E-08	0.358782843	0.42	0.926
Rrad	2.14E-24	0.358186587	0.212	0.691
Akap8l	8.74E-10	0.356674308	0.117	0.336
Gpatch8	2.48E-13	0.356543099	0.144	0.435
4932438A13Rik	6.64E-14	0.356293471	0.163	0.48
Raf1	2.28E-18	0.355167362	0.182	0.569
Tead3	2.57E-16	0.354949134	0.125	0.447
Cacna1c	2.68E-10	0.352865921	0.106	0.325
Nsmf	4.88E-07	0.350732376	0.083	0.233
Esyt2	3.38E-18	0.35014732	0.322	0.869
Fnbp4	6.54E-07	0.34997416	0.114	0.279
Rbfox2	7.59E-22	0.348906284	0.216	0.699
Arfip2	1.11E-07	0.348742064	0.098	0.265
Mycbp2	2.49E-14	0.348706317	0.254	0.657
Camkk2	9.24E-14	0.348580582	0.083	0.331
Mia3	4.18E-16	0.348352853	0.163	0.504
Ppp1r12b	1.91E-06	0.348310619	0.424	0.938

Ints7	0.000453657	0.348132833	0.064	0.153
Ubn2	2.88E-15	0.34794611	0.189	0.538
Gadd45g	6.96E-12	0.347729099	0.345	0.778
Synj2	2.47E-06	0.346648736	0.076	0.21
Ppp1r12c	2.92E-18	0.346301244	0.292	0.786
Phf3	1.96E-19	0.344627387	0.178	0.573
Smarcd2	0.000736913	0.343967862	0.095	0.195
Camk2d	4.92E-21	0.343892218	0.284	0.813
Fosl2	9.71E-14	0.343314533	0.159	0.464
Chd1	1.69E-09	0.342425956	0.114	0.325
Smg1	6.12E-12	0.342366469	0.186	0.486
Clasp2	1.23E-15	0.342140132	0.159	0.483
Gtf3c2	3.60E-09	0.339875775	0.114	0.313
Trio	5.62E-20	0.339157904	0.205	0.635
Dlgap4	1.32E-20	0.33802817	0.189	0.618
Ccdc82	1.11E-14	0.337414948	0.136	0.434
Hnrnpdl	4.91E-17	0.337238544	0.303	0.795
Guf1	5.78E-08	0.336213612	0.08	0.239
Ptprs	8.38E-24	0.334826114	0.227	0.715
Sbf2	3.06E-11	0.333876129	0.114	0.349
Aff4	4.98E-17	0.333864873	0.303	0.797
Carmn	4.93E-19	0.333817304	0.174	0.565
Nipbl	4.81E-21	0.333322458	0.231	0.702
Malt1	2.35E-07	0.333176733	0.072	0.221
Carns1	1.50E-07	0.332214033	0.095	0.26
Nrbp1	9.35E-19	0.33173181	0.144	0.496
Chd6	2.49E-18	0.33165519	0.295	0.826
Rasl12	2.09E-20	0.331440164	0.273	0.806
Arglu1	1.85E-22	0.331403012	0.246	0.737
Gpcpd1	3.44E-09	0.330641224	0.102	0.294
Nudt13	3.14E-08	0.330545801	0.091	0.266
Mylk2	5.50E-05	0.33051636	0.057	0.155
Serpine1	1.46E-10	0.329957907	0.333	0.745
Lsp1	0.003523253	0.32995205	0.098	0.184
Samd9l	1.27E-05	0.329857644	0.11	0.25
Lama4	1.29E-21	0.32964107	0.22	0.69
Otud4	1.89E-09	0.329384248	0.091	0.281
Klf6	6.14E-06	0.329174104	0.436	0.903
Kcnq1ot1	1.22E-12	0.329059098	0.136	0.396
Tef	1.81E-23	0.328984516	0.231	0.735
Ankrd44	1.41E-05	0.327210461	0.106	0.246
Mfn1	0.000333436	0.326411071	0.076	0.168
Papd5	2.40E-06	0.326321073	0.095	0.24
Usp15	5.69E-14	0.32612512	0.117	0.389

Srsf6	6.41E-19	0.32535041	0.178	0.565
Zeb2	7.24E-15	0.32517788	0.148	0.46
Irs2	4.22E-14	0.324856646	0.167	0.479
Zkscan3	2.24E-15	0.324573256	0.095	0.367
Inppl1	2.27E-08	0.324545504	0.087	0.258
Gm17056	0.008692156	0.323821466	0.053	0.109
Dnajc13	6.47E-07	0.323642135	0.098	0.252
Traf7	8.61E-16	0.323523564	0.098	0.378
Kif13a	4.20E-24	0.322913358	0.152	0.575
Thrb	9.09E-21	0.322314747	0.159	0.555
Arid1a	7.78E-14	0.32155598	0.159	0.458
Errfi1	3.81E-17	0.321067153	0.201	0.573
Tsc1	0.003682042	0.320469707	0.076	0.148
Chd2	4.35E-12	0.320249152	0.129	0.386
Itga7	2.04E-21	0.319886319	0.163	0.576
Supt5	1.08E-16	0.318749254	0.182	0.543
Tmem176b	0.118252721	0.318631312	0.08	0.119
Camk2g	1.32E-25	0.317130028	0.182	0.639
Ogt	2.24E-20	0.317107815	0.182	0.596
Ankrd11	6.29E-21	0.317054064	0.284	0.79
Ets1	8.65E-05	0.3165592	0.102	0.22
Fam71a	7.10E-05	0.316074814	0.057	0.153
Arid5a	6.12E-09	0.315764846	0.144	0.365
Lama5	2.13E-16	0.313534676	0.129	0.443
Luc7l	3.86E-12	0.313499374	0.117	0.368
Pdlim3	9.13E-06	0.313375889	0.443	0.937
Creb5	1.32E-16	0.312997081	0.22	0.622
Kmt2c	1.26E-18	0.31286772	0.17	0.556
Fubp1	9.62E-16	0.312412231	0.174	0.514
Npc2	1.51E-27	0.311725297	0.178	0.663
Ttc14	2.73E-18	0.311685165	0.17	0.548
Macf1	6.99E-05	0.310078786	0.473	0.937
Mrto4	3.33E-08	0.310009794	0.095	0.262
Peg3	1.42E-16	0.3099377	0.114	0.42
Ehbp1l1	6.29E-26	0.307927377	0.201	0.676
Dnm2	2.45E-08	0.307633112	0.117	0.305
C2cd2	1.82E-16	0.307431601	0.098	0.386
Marf1	5.85E-17	0.307339267	0.117	0.422
Map3k5	3.71E-08	0.307267665	0.117	0.309
Rexo1	1.51E-10	0.306952708	0.091	0.301
Ppfibp1	1.09E-20	0.306773022	0.125	0.481
Stk38	2.46E-12	0.306262959	0.114	0.365
Gm47283	2.54E-08	0.306157483	0.117	0.309
Rsrc2	8.77E-18	0.306008961	0.261	0.716

Dnajc7	5.26E-11	0.305935195	0.098	0.319
Mtch2	4.95E-24	0.305882529	0.182	0.62
Sec24b	1.59E-08	0.305755225	0.091	0.267
Anapc5	2.74E-24	0.305668903	0.216	0.701
Pcgf3	1.77E-09	0.30501687	0.087	0.278
Tcf25	7.67E-17	0.30497629	0.318	0.824
Xirp1	2.00E-15	0.303902193	0.102	0.38
Klf15	6.14E-20	0.303358495	0.117	0.475
Hey2	1.27E-22	0.302742917	0.133	0.529
Lrrfip1	2.37E-05	0.302681728	0.462	0.944
Nsd3	1.00E-13	0.302039064	0.163	0.475
Slc39a1	6.84E-22	0.30152022	0.182	0.612
Btg2	0.636312392	0.301325844	0.606	0.925
Mkln1	2.40E-21	0.30118326	0.159	0.56
Tet3	1.25E-14	0.301078893	0.106	0.382
Mib1	6.96E-22	0.300956881	0.129	0.502
Odf2	4.09E-06	0.300757604	0.095	0.239
Scarf2	1.12E-15	0.300474062	0.136	0.459
Mta1	5.77E-13	0.300319507	0.102	0.351
Ryr2	1.45E-17	0.30010929	0.133	0.465
Zw10	4.33E-05	0.299537855	0.061	0.165
Leng8	8.17E-15	0.299053151	0.155	0.477
Wsb1	5.79E-08	0.298785689	0.152	0.356
Ptprz1	1.75E-15	0.298688231	0.277	0.728
Ptp4a1	2.38E-25	0.298248163	0.167	0.606
Clip1	6.68E-22	0.298172076	0.258	0.762
Trp53inp1	1.88E-19	0.298095357	0.189	0.592
Nr4a3	0.000582466	0.297200782	0.057	0.134
Immt	1.44E-22	0.296783553	0.152	0.546
Hnrnpl	1.44E-26	0.296722354	0.182	0.646
Cd44	0.000141316	0.296532427	0.08	0.183
Stx1a	2.70E-08	0.296279612	0.091	0.27
Lrrc45	0.000173888	0.295890582	0.087	0.191
Rgl2	0.000194715	0.295869805	0.076	0.176
Tmem131l	6.88E-05	0.295818911	0.087	0.201
Camk1	1.30E-25	0.295681012	0.189	0.656
Ehd4	4.47E-17	0.295443666	0.182	0.553
Ppp4r3a	4.90E-14	0.295185109	0.117	0.389
Gm26722	2.98E-17	0.294916534	0.087	0.372
Cables1	0.000291633	0.294450723	0.064	0.155
2010111I01Rik	8.06E-12	0.294223712	0.356	0.873
R3hdm1	6.61E-14	0.293814684	0.182	0.506
Rfng	0.000273586	0.293774382	0.061	0.149
Sdccag3	9.38E-08	0.293729987	0.08	0.239

Irf2bp2	1.61E-21	0.293679194	0.258	0.734
Clk2	9.00E-05	0.293117103	0.061	0.157
Rc3h2	3.70E-16	0.291988101	0.144	0.465
Prpf38b	2.31E-22	0.291947466	0.212	0.659
Csnk1d	8.20E-15	0.291668405	0.117	0.405
Phf21a	3.33E-11	0.291582783	0.095	0.306
Plekhg2	1.83E-09	0.29154545	0.08	0.26
Brd8	1.56E-13	0.291510308	0.136	0.411
Zfp638	1.71E-12	0.291337565	0.189	0.502
Hmg20b	1.07E-23	0.290789239	0.182	0.617
Brd9	7.07E-16	0.290780447	0.133	0.439
Erc1	3.11E-15	0.290358647	0.117	0.408
Hmgb2	1.91E-14	0.290012208	0.11	0.383
Cyth3	2.58E-25	0.289840533	0.212	0.701
Spg11	0.00123669	0.289532196	0.042	0.108
Nsd1	2.53E-17	0.288135251	0.167	0.525
Lrrcc1	5.84E-16	0.288093146	0.117	0.414
Ulk1	3.19E-10	0.287212185	0.072	0.256
Chuk	3.19E-06	0.287008812	0.098	0.243
Psme4	7.58E-16	0.286827137	0.186	0.529
Vps37b	8.66E-10	0.285927466	0.08	0.266
Ralgapa2	1.13E-13	0.285079899	0.064	0.296
Xpo6	1.03E-06	0.284932324	0.091	0.237
Bicd1	2.18E-23	0.284778801	0.121	0.509
Ttc8	0.000279887	0.284395715	0.076	0.171
Usp34	1.21E-20	0.283457787	0.223	0.676
Per1	2.34E-13	0.283277936	0.148	0.447
Wdr48	5.28E-09	0.283255513	0.087	0.271
Jmjd1c	7.50E-14	0.283180703	0.17	0.494
Vasp	3.72E-26	0.28284799	0.246	0.77
Srsf7	1.09E-17	0.282615934	0.148	0.486
Adgre5	4.94E-18	0.282601036	0.152	0.512
Unk	0.005586756	0.282217511	0.049	0.108
Npr2	5.56E-12	0.282011767	0.091	0.316
Mthfr	9.23E-05	0.281938837	0.053	0.145
Itga5	1.91E-15	0.281890413	0.197	0.553
Parp8	9.76E-08	0.281795363	0.098	0.27
Usp19	3.71E-16	0.281703673	0.133	0.445
Trrap	4.72E-10	0.281470258	0.068	0.252
Arhgef10l	1.45E-18	0.280636725	0.095	0.407
Hdac5	4.62E-22	0.280188962	0.163	0.566
Thap3	1.10E-11	0.280165963	0.098	0.323
Mtss1	8.03E-17	0.279803591	0.174	0.528
Slc25a51	3.15E-09	0.27726207	0.106	0.299

Fam193b	1.12E-05	0.277078166	0.076	0.199
Nop56	7.48E-14	0.277032944	0.136	0.421
Gna13	4.85E-10	0.276902056	0.095	0.29
Trim2	7.97E-05	0.276652717	0.045	0.134
Mdm4	1.11E-16	0.276276157	0.117	0.425
Srsf10	3.38E-22	0.275544619	0.231	0.71
Atf7ip	4.75E-11	0.275413615	0.136	0.38
Map4k2	0.000541474	0.27525438	0.053	0.13
Akap8	3.74E-09	0.274935801	0.102	0.296
Wnk1	3.17E-16	0.274742371	0.303	0.817
Rmnd1	9.46E-06	0.274704204	0.061	0.174
Med25	7.16E-12	0.274667362	0.098	0.328
Llph	1.16E-15	0.27464093	0.129	0.426
Gtpbp2	8.35E-11	0.274391737	0.121	0.353
Rnf111	1.19E-10	0.273995997	0.091	0.295
Git2	8.04E-08	0.273570247	0.121	0.305
Hk2	2.26E-13	0.273248016	0.133	0.411
Abcc1	6.26E-21	0.272936822	0.144	0.521
Prpf40b	5.72E-06	0.272847004	0.053	0.166
Napg	4.20E-17	0.272554921	0.11	0.415
Ascc3	3.22E-08	0.272380577	0.121	0.313
Rab8b	0.001950546	0.272345491	0.053	0.121
Wdr26	2.16E-20	0.271871812	0.178	0.574
Cspg4	7.78E-10	0.271229859	0.402	0.924
Mtus1	9.89E-23	0.270790102	0.174	0.608
Mark2	1.62E-13	0.270691729	0.129	0.399
Cdk12	2.63E-14	0.270527731	0.152	0.458
Mknk2	3.84E-19	0.26983006	0.163	0.542
Coq2	2.83E-15	0.26976459	0.102	0.379
Vps13d	1.71E-11	0.269665691	0.076	0.284
Dmwd	2.33E-13	0.267589928	0.095	0.347
Dmtf1	1.27E-06	0.267531269	0.087	0.23
Zc3h7a	2.69E-11	0.267425112	0.133	0.386
Nfe2l2	6.91E-22	0.266394019	0.17	0.585
Rcor3	3.94E-14	0.266325898	0.098	0.361
Rnf25	0.002671143	0.266241446	0.049	0.114
Kansl1l	2.26E-11	0.266173908	0.106	0.336
Sat1	4.77E-13	0.266098854	0.371	0.879
Slc25a36	1.10E-11	0.266092143	0.106	0.345
Gramd1a	2.80E-05	0.265368887	0.087	0.209
Inpp4a	3.42E-21	0.265362495	0.117	0.478
Gcn1l1	2.69E-12	0.265345979	0.098	0.334
Iqgap1	5.61E-15	0.264055855	0.33	0.86
Ric1	3.32E-09	0.263838496	0.076	0.255

Fbxl20	1.25E-16	0.263635437	0.098	0.386
Stk38l	1.82E-24	0.262350288	0.17	0.602
Gga2	2.02E-16	0.262277538	0.102	0.397
Itgb3	5.95E-16	0.262175546	0.095	0.38
Emc1	3.96E-12	0.262000235	0.102	0.339
Ifrd1	5.38E-24	0.261710416	0.193	0.652
Rps6kb1	1.90E-13	0.261460926	0.114	0.376
Ubp1	7.23E-17	0.261095057	0.155	0.496
Phrf1	5.33E-07	0.261066398	0.08	0.222
Rela	1.58E-13	0.26032683	0.14	0.428
Brd2	1.21E-20	0.260231575	0.258	0.745
Zbtb24	0.03585948	0.260060797	0.061	0.106
Tyw1	6.65E-05	0.260018319	0.057	0.15
Hmg20a	3.28E-08	0.259838315	0.076	0.239
Insr	7.32E-20	0.259301634	0.144	0.52
Atxn2l	2.77E-14	0.25928316	0.121	0.404
Sorbs2	4.87E-25	0.259196659	0.174	0.627
Coq10b	4.54E-24	0.258856191	0.235	0.727
Yap1	1.26E-19	0.258807076	0.095	0.415
Smad7	1.11E-19	0.258527851	0.288	0.794
Lims2	6.12E-17	0.25827068	0.333	0.883
R3hdm2	3.99E-26	0.258193269	0.155	0.597
Tra2a	7.05E-22	0.257936092	0.258	0.755
Bhlhe40	7.99E-08	0.257541196	0.057	0.199
Ulk4	0.017067446	0.257481486	0.057	0.108
Gm26699	0.000623495	0.257445015	0.049	0.125
Mecp2	2.39E-20	0.256736875	0.129	0.479
Adcy6	7.71E-12	0.256591314	0.106	0.343
Dock7	2.53E-20	0.256370464	0.106	0.449
Dnaja1	7.72E-36	0.256317968	0.254	0.856
Slc12a4	9.12E-16	0.255779225	0.117	0.409
Fry	2.71E-31	0.255669897	0.216	0.78
Trip11	3.62E-21	0.255012239	0.197	0.625
Pfkp	3.16E-26	0.255007973	0.197	0.685
Ankrd27	2.18E-08	0.254843147	0.064	0.217
Sec24d	1.80E-29	0.254001486	0.155	0.632
Arhgef11	9.50E-07	0.253946529	0.076	0.217
Fcho2	2.97E-23	0.253618919	0.163	0.587
Ap3m1	1.32E-14	0.253610465	0.117	0.4
Sf3b3	1.02E-14	0.252913599	0.08	0.333
Tnxb	1.65E-22	0.25250686	0.182	0.62
Dab2	0.001186499	0.251360935	0.057	0.132
Agap3	8.10E-08	0.251254383	0.091	0.26
Ints13	1.89E-06	0.251034294	0.053	0.175

Tubgcp3	7.86E-07	0.250421603	0.072	0.208
Apol6	0.000354078	0.250374622	0.042	0.118

"gene":the name of each differentially expressed gene.
"*p*_val": *p* value of significance test. If there are too many decimal places, 0 will be displayed;
"avg_logFC": fold change of gene average expression level.
"pct.1": the proportion of cells expressing this gene of particular cluster.
"pct.2": the proportion of cells expressing this gene of the rest subpopulations.
