# Supplementary material for: A regulator of G protein signaling 5 marked subpopulation of vascular smooth muscle cells is lost during vascular disease
Source: PLoS One. 2022 Mar 23;17(3):e0265132. doi: 10.1371/journal.pone.0265132 (PMC8942229; doi:10.1371/journal.pone.0265132)
Supplement: S9 File — (PDF) [file pone.0265132.s012.pdf]

Differentially expressed genes in VSMC_9 cluster

gene	p_val	avg_logFC	pct.1	pct.2
Sncg	4.96E-179	2.208468521	0.929	0.118
Pln	4.05E-255	1.913883737	0.832	0.058
Igfbp2	5.20E-111	1.80830225	0.655	0.084
Crip1	6.16E-63	1.557794553	1	0.945
Sorbs2	1.53E-63	1.506094096	1	0.609
Igfbp4	1.23E-90	1.489788046	0.752	0.136
S100a4	1.55E-36	1.467859324	0.894	0.649
Pappa2	0	1.39898034	0.327	0
Epas1	1.58E-64	1.37609812	0.973	0.478
Mgp	8.80E-22	1.342344283	1	1
Palld	1.21E-52	1.285974522	0.956	0.568
Mustn1	1.89E-48	1.174185777	1	0.782
Prss23	2.21E-40	1.111962019	0.947	0.67
Notch3	3.63E-81	1.097771882	0.876	0.231
Serpini1	3.28E-36	1.058180232	0.903	0.644
Pdgfc	3.99E-55	0.987165908	0.761	0.24
Nrgn	1.58E-24	0.972515721	0.372	0.101
C1s1	9.81E-98	0.896681034	0.628	0.086
Acan	6.08E-25	0.86283809	0.575	0.231
Sparcl1	3.89E-18	0.86243596	0.894	0.681
Phex	3.47E-62	0.851486072	0.469	0.071
Crim1	5.69E-37	0.847058068	0.973	0.741
Gdf10	6.28E-230	0.826000656	0.549	0.024
Mob2	6.08E-34	0.816249855	0.894	0.584
Gm37800	0	0.802662047	0.416	0.002
Pdgfa	8.91E-22	0.798774687	0.92	0.777
Gja4	2.31E-55	0.793756544	0.602	0.132
Plpp3	8.48E-25	0.791132425	0.858	0.644
Gng11	9.41E-38	0.787853808	0.85	0.433
Utrn	3.32E-35	0.784744274	0.991	0.864
Jag1	2.63E-32	0.753694278	0.69	0.282
Kalrn	6.48E-34	0.748048999	0.743	0.34
Cfh	8.36E-24	0.728645945	0.814	0.424
Aspn	8.81E-11	0.718113684	0.425	0.212
Tnfrsf11b	1.83E-22	0.717471368	0.451	0.149
Tmem176b	1.85E-74	0.713969448	0.655	0.111
Slc6a6	1.10E-25	0.688910992	0.867	0.647
Phlda3	7.53E-30	0.678251241	0.708	0.298
Nid1	3.62E-52	0.662737874	0.549	0.111
Angpt2	1.74E-28	0.654639873	0.655	0.267
Crlf1	6.04E-55	0.641465269	0.425	0.064

Rasal2	4.61E-30	0.631082017	0.761	0.365
Ecm1	1.07E-17	0.627995532	0.947	0.866
Thbs1	5.43E-70	0.613539275	0.345	0.032
S100a6	3.25E-27	0.599912871	1	0.961
Htr1b	6.15E-15	0.599182519	0.673	0.448
Serping1	1.12E-21	0.598328238	0.814	0.553
Plvap	6.08E-11	0.598002297	0.195	0.056
Nov	1.23E-15	0.592191418	0.965	0.916
Pianp	2.27E-263	0.591176465	0.451	0.013
Gldn	1.15E-24	0.584830703	0.566	0.226
Crispld1	1.52E-14	0.583099575	0.407	0.173
Synm	2.73E-22	0.574107209	0.814	0.5
Tbx2	6.24E-83	0.557156799	0.487	0.058
Perp	2.50E-17	0.553090516	0.894	0.701
Limd1	5.39E-17	0.549582916	0.752	0.472
Ndrg1	8.39E-20	0.545122932	0.77	0.447
Pacsin2	1.40E-17	0.53686499	0.832	0.621
P2rx1	4.30E-26	0.533985662	0.558	0.193
Gucy1a1	1.02E-16	0.528740836	0.894	0.823
Nrp1	6.08E-12	0.528582418	0.531	0.293
Tspo	2.97E-15	0.524783582	0.929	0.872
Pgf	1.15E-89	0.523369811	0.434	0.042
Wtip	1.19E-17	0.521684052	0.947	0.815
Myh9	1.90E-13	0.5200907	0.841	0.703
Arhgdib	2.37E-47	0.518009475	0.434	0.075
Olfml2a	1.49E-34	0.515548719	0.451	0.109
1500009L16Rik	1.73E-19	0.515251305	0.531	0.232
Cd248	5.53E-32	0.515137516	0.451	0.117
Ntn4	5.32E-21	0.513669146	0.54	0.214
9930111J21Rik2	4.60E-55	0.512666803	0.398	0.055
Cd109	5.00E-27	0.507471275	0.602	0.224
Ptn	1.02E-130	0.50444232	0.23	0.007
Gprc5c	0	0.501257803	0.416	0.003
Zfp945	1.93E-12	0.500859974	0.469	0.235
Cd82	1.40E-58	0.498255421	0.54	0.097
Esam	1.49E-24	0.494424081	0.593	0.227
Bmp2	1.72E-29	0.490271517	0.531	0.159
Lamb2	1.07E-22	0.485541516	0.991	0.958
Cd200	5.37E-17	0.48487098	0.973	0.937
Ftl1	1.17E-21	0.482864239	0.947	0.869
Tsc22d3	6.17E-15	0.482601965	0.584	0.309
Igfbp7	4.94E-31	0.480770087	1	0.991
C1ra	3.86E-48	0.478870735	0.522	0.107
Mast4	3.07E-16	0.478105627	0.832	0.623

Galnt15	5.06E-14	0.477986216	0.407	0.17
Ckb	2.56E-11	0.477270617	0.903	0.731
Hes1	8.46E-12	0.476978938	0.628	0.403
Gas6	1.82E-06	0.466672363	0.858	0.893
Rnf152	1.34E-40	0.465631564	0.496	0.109
Tgfb3	2.63E-12	0.464889571	0.655	0.409
Ldlrad3	2.93E-13	0.46340568	0.558	0.289
Timp4	1.48E-10	0.463374939	0.425	0.202
Lamc3	1.60E-06	0.461002283	0.336	0.182
Pde1a	1.09E-18	0.454776509	0.575	0.251
Arpc1b	3.42E-10	0.454258027	0.929	0.854
Blmh	1.12E-13	0.452179285	0.708	0.475
Scn1b	1.26E-20	0.45041911	0.637	0.297
Gm13861	1.72E-12	0.448274165	0.487	0.241
Fxyd5	1.42E-11	0.445528591	0.487	0.247
Trim2	2.23E-30	0.439309181	0.469	0.127
Nes	0.001016491	0.438159396	0.619	0.59
Mertk	1.68E-13	0.437733386	0.681	0.432
Jcad	4.74E-17	0.431838846	0.549	0.254
Zeb2	7.80E-14	0.430885799	0.699	0.448
Uba2	1.26E-09	0.428337451	0.832	0.712
Tmem204	2.47E-79	0.425375495	0.389	0.037
Ankrd1	1.90E-09	0.425237974	0.336	0.148
Ptp4a3	2.78E-08	0.418224092	0.735	0.571
Cyb5r3	2.47E-11	0.416944616	0.92	0.899
Art3	1.61E-10	0.415074874	0.752	0.488
Zfhx3	3.65E-15	0.413268042	0.973	0.891
Col14a1	4.83E-05	0.41214336	0.451	0.32
Lims1	1.36E-10	0.411388192	0.779	0.63
Anxa1	8.28E-09	0.410904799	0.903	0.789
Crip2	2.69E-16	0.40884154	0.92	0.854
Emp3	2.60E-08	0.405042508	0.947	0.88
Raph1	4.95E-21	0.402328091	0.522	0.196
Isyna1	2.85E-11	0.402149004	0.558	0.34
F2r	1.97E-09	0.399602161	0.558	0.35
Cavin3	3.87E-15	0.399065663	0.956	0.889
Kcna5	2.80E-109	0.397530193	0.336	0.019
Sdc2	2.18E-13	0.395541561	0.876	0.691
Kitl	2.31E-12	0.390052913	0.602	0.34
Tmem176a	3.19E-52	0.389383664	0.407	0.059
Ebf1	1.33E-11	0.388847884	0.894	0.809
Timp3	1.39E-07	0.387189079	0.982	0.972
Atf5	2.99E-08	0.385926165	0.611	0.421
Ednra	1.06E-71	0.384983888	0.319	0.027

Inhba	7.92E-10	0.383451973	0.761	0.603
Serpinb1a	4.13E-80	0.382034374	0.301	0.022
Ctdspl	5.65E-11	0.37983531	0.699	0.501
Trim47	1.08E-14	0.378564484	0.832	0.618
Svil	2.94E-09	0.3755715	0.912	0.788
Ptges3l	1.11E-10	0.373747041	0.504	0.281
Sfxn1	2.67E-11	0.373466104	0.531	0.3
Slc7a2	5.86E-17	0.371272476	0.513	0.214
Atp2b1	4.76E-11	0.370703445	0.779	0.647
Col8a1	7.32E-36	0.36958695	0.319	0.052
Plekhg2	4.52E-13	0.365757545	0.496	0.252
Apbb2	5.25E-13	0.365411011	0.478	0.225
Dhx58os	2.06E-226	0.364560881	0.301	0.006
Nr2f2	1.99E-10	0.358164554	0.752	0.536
Abr	1.20E-13	0.358151692	0.496	0.231
Phldb2	1.44E-10	0.356568203	0.876	0.741
Cd151	1.63E-13	0.35638899	0.92	0.819
Cd34	1.75E-23	0.355672175	0.265	0.052
Tinagl1	1.16E-07	0.352247425	0.858	0.763
Lifr	1.05E-08	0.352152341	0.575	0.362
Rtn4rl1	5.59E-41	0.351612954	0.274	0.035
Rgs7bp	1.80E-09	0.349901326	0.867	0.804
Actn4	2.48E-16	0.347914506	0.982	0.951
Myl12b	8.81E-10	0.347205437	0.805	0.649
Mfge8	9.78E-15	0.347014192	0.991	0.966
Mef2c	2.27E-09	0.345170278	0.956	0.883
Myo1e	1.48E-08	0.342993426	0.558	0.368
Cbx6	6.46E-11	0.341988145	0.894	0.707
Vat1	1.17E-08	0.341740106	0.602	0.407
Dag1	5.12E-13	0.341097903	0.894	0.777
Sulf1	4.41E-05	0.339821358	0.796	0.754
Slit3	5.17E-11	0.339724401	0.735	0.525
Tmod3	1.52E-12	0.337990946	0.637	0.369
Tpm4	9.37E-10	0.33487475	0.796	0.626
Prkar1a	4.86E-11	0.334246675	0.965	0.871
Fam81a	7.67E-07	0.332956554	0.513	0.353
Rhoa	3.35E-15	0.331687352	0.991	0.941
Abhd2	3.06E-12	0.331299739	0.513	0.271
Epn2	5.86E-09	0.33015981	0.788	0.637
Hipk1	2.88E-10	0.327959204	0.788	0.632
Agrn	1.24E-10	0.324724404	0.575	0.344
Dlc1	9.83E-11	0.323489502	0.77	0.526
Cmklr1	3.33E-54	0.319807538	0.416	0.06
Fth1	4.44E-13	0.319606013	1	0.995

Tns2	7.15E-09	0.319595227	0.832	0.669
Col4a4	2.42E-19	0.317235879	0.363	0.103
Snx9	2.08E-07	0.315349865	0.602	0.429
Aoc3	1.97E-09	0.315023789	0.912	0.834
Npy1r	1.06E-07	0.314058348	0.876	0.783
Akap12	1.54E-19	0.312948722	0.381	0.117
Knop1	2.98E-16	0.312301823	0.54	0.237
Cspg4	6.75E-12	0.311297304	0.973	0.907
Gpx1	4.52E-08	0.308581858	0.982	0.91
Rpa1	2.53E-06	0.308074387	0.442	0.276
Anxa2	9.55E-08	0.305834008	0.894	0.746
Rapgef5	6.28E-10	0.305217645	0.593	0.366
Anxa5	4.44E-09	0.304822571	0.92	0.873
Cacna2d1	1.50E-05	0.29748759	0.805	0.749
Ankrd28	1.02E-19	0.297156099	0.345	0.097
Calm1	2.63E-11	0.296453607	0.991	0.955
Ppp1r14b	1.91E-06	0.296102722	0.54	0.393
Endod1	5.59E-09	0.295740012	0.832	0.692
Rgs17	4.84E-08	0.294666425	0.23	0.088
Atpif1	1.59E-08	0.29433488	0.903	0.84
Dusp5	1.23E-11	0.292504778	0.221	0.065
Trabd2b	7.41E-06	0.292005583	0.628	0.497
Parm1	5.06E-09	0.291059279	0.513	0.298
Tmem51	2.26E-40	0.29088222	0.319	0.047
Gprc5b	3.09E-111	0.290864652	0.265	0.011
Kras	2.95E-09	0.289952017	0.611	0.416
Pear1	3.90E-14	0.28741341	0.469	0.204
Map7d2	1.16E-08	0.287142869	0.504	0.29
Dclk1	3.78E-34	0.285647042	0.239	0.032
Pde3a	3.97E-07	0.285589128	0.903	0.819
Chd4	1.37E-05	0.285122254	0.867	0.773
Fhl2	1.40E-20	0.284463783	0.292	0.07
Gm13889	0.000150207	0.283591845	0.867	0.779
Junb	0.04447257	0.28325274	0.788	0.761
Mapre2	5.41E-09	0.28310337	0.717	0.513
Mprip	7.04E-09	0.282737848	0.832	0.692
Klf2	4.44E-05	0.28116886	0.85	0.689
Arhgap1	3.71E-07	0.279898442	0.487	0.308
Cdk14	1.79E-19	0.279841727	0.319	0.087
Klf7	9.17E-08	0.279567599	0.814	0.626
Tacc1	4.32E-08	0.278965695	0.885	0.817
Csrp1	3.25E-09	0.277637471	0.991	0.968
Lhfp	1.55E-09	0.277263048	0.982	0.916
Eea1	2.91E-07	0.276924533	0.805	0.699

Iqgap1	4.88E-08	0.2763174	0.912	0.844
Capg	8.38E-10	0.274836386	0.345	0.154
Tm4sf1	7.73E-08	0.274635464	0.973	0.956
Stk32b	2.60E-102	0.274360257	0.265	0.013
Gpi1	1.50E-07	0.274342084	0.885	0.82
Lgalsl	9.65E-06	0.274097244	0.779	0.658
Sdc3	3.46E-36	0.273445962	0.319	0.051
Atp2c1	8.38E-08	0.27336112	0.504	0.321
Twist1	8.59E-06	0.27292411	0.434	0.275
Mlph	5.73E-79	0.271978425	0.23	0.013
Soat1	4.93E-06	0.271263965	0.389	0.23
Zfp40	1.25E-07	0.270916691	0.177	0.061
Mrps6	1.43E-05	0.270858527	0.522	0.367
Foxs1	4.58E-09	0.270503842	0.301	0.131
Il1r1	1.93E-08	0.26883724	0.46	0.257
Cdkn2a	7.92E-219	0.268283516	0.186	0.001
Ccnd1	1.13E-28	0.26796745	0.168	0.019
Mxd4	1.75E-05	0.267850942	0.841	0.728
Eng	6.75E-08	0.267106243	0.46	0.264
Spry4	1.17E-12	0.266852002	0.274	0.088
Tcf4	4.47E-07	0.266748877	0.681	0.495
Mindy2	1.03E-08	0.266651532	0.469	0.258
Epb41l2	2.09E-06	0.266450914	0.549	0.393
Cox4i2	4.85E-10	0.26631322	0.265	0.098
Ehd4	5.11E-07	0.266196156	0.726	0.539
Nme1	2.07E-08	0.26615264	0.664	0.455
Susd5	2.01E-07	0.26558579	0.938	0.902
Prkg1	4.07E-05	0.265566315	0.478	0.346
Fzd1	5.78E-09	0.265556953	0.708	0.491
Ptpn9	1.12E-07	0.264810986	0.487	0.29
Fam129b	2.43E-06	0.26455679	0.584	0.441
Col4a3	5.52E-19	0.262790904	0.248	0.055
Tsc22d1	0.000772524	0.262623009	0.929	0.888
Armc3	1.93E-65	0.261372272	0.265	0.021
Lama5	9.93E-07	0.260625183	0.575	0.431
Calm3	8.66E-07	0.260022805	0.885	0.793
Chst11	1.50E-49	0.259956038	0.265	0.027
Egr2	4.02E-22	0.259593915	0.204	0.034
Ccdc88a	2.35E-07	0.259331542	0.717	0.561
Pdlim2	6.65E-06	0.259121008	0.673	0.51
Pea15a	1.61E-06	0.258918654	0.637	0.473
Cpt1a	6.86E-10	0.258575091	0.478	0.251
Ywhag	4.09E-06	0.257531708	0.637	0.503
Chst15	3.30E-25	0.257445003	0.195	0.028

Chn2	4.80E-46	0.257396871	0.301	0.037
Ate1	1.44E-06	0.257228366	0.522	0.356
Ryr2	0.001150768	0.255893671	0.54	0.454
Cdh11	3.23E-20	0.254900373	0.283	0.066
Msn	2.61E-05	0.25459512	0.965	0.91
Enpep	1.77E-09	0.252464143	0.265	0.101
Klhdc8b	2.77E-08	0.251532328	0.566	0.348
Ptma	2.19E-11	0.250470043	0.982	0.979
Hrct1	1.26E-08	0.250333053	0.354	0.167

“gene”:the name of each differentially expressed gene.
“*p*_val”: *p* value of significance test. If there are too many decimal places, 0 will be displayed;
“avg_logFC”: fold change of gene average expression level.
“pct.1”: the proportion of cells expressing this gene of particular cluster.
“pct.2”: the proportion of cells expressing this gene of the rest subpopulations.
